# Unraveling Lennox–Gastaut Syndrome: From Molecular Pathogenesis to Precision Diagnosis and Targeted Therapy Evolving Therapeutic Strategies

**DOI:** 10.3390/ijms27031382

**Published:** 2026-01-30

**Authors:** Ji-Hoon Na, Young-Mock Lee

**Affiliations:** Department of Pediatrics, Gangnam Severance Hospital, Yonsei University College of Medicine, Seoul 135-720, Republic of Korea; jhnamd83@yuhs.ac

**Keywords:** Lennox–Gastaut syndrome, developmental and epileptic encephalopathy, thalamocortical network, drop seizures, cannabidiol, precision medicine, electroencephalography, neuromodulation, ketogenic diet

## Abstract

Lennox–Gastaut syndrome (LGS) is a rare and severe developmental and epileptic encephalopathy characterized by multiple drug-resistant seizure types, mandatory tonic seizures, cognitive and behavioral impairment, and distinctive electroencephalographic features, including slow spike–wave discharges and generalized paroxysmal fast activity. Despite decades of therapeutic advances, LGS remains associated with profound lifelong disability and the absence of a single disease-defining molecular mechanism. Recent advances in genetics, neurophysiology, and network neuroscience have reframed LGS as a convergent network encephalopathy, in which diverse genetic, structural, metabolic, immune, and acquired insults funnel into shared molecular hubs, leading to thalamocortical network dysfunction. This framework helps explain the limited efficacy of purely syndrome-based treatments. This review synthesizes current evidence on electroclinical phenotyping, molecular and network pathogenesis, and contemporary diagnostic workflows and proposes a molecule-to-precision-therapy framework for LGS. We critically appraise pharmacologic, dietary, surgical, and neuromodulatory therapies, emphasizing drop seizures as a major driver of morbidity. Among available treatments, cannabidiol shows the most consistent and clinically meaningful efficacy for drop seizures, with benefits extending beyond seizure counts to seizure-free days and caregiver-relevant outcomes. Finally, we highlight key gaps and future directions, including etiology-stratified trials, network-guided interventions, and outcome measures that capture long-term developmental and quality-of-life impacts.

## 1. Introduction

Lennox–Gastaut syndrome (LGS) is a rare and severe form of developmental and epileptic encephalopathy (DEE) that typically manifests in early childhood, most often between the ages of 2 and 5 years [1,2,3,4]. It is characterized by a triad of symptoms comprising multiple seizure types, including tonic and atonic seizures; cognitive impairment; and a distinctive electroencephalogram (EEG) pattern marked by slow spike-and-wave (SSW) discharges accompanied by generalized paroxysmal fast activity (GPFA) [5,6,7]. LGS frequently evolves from other epileptic syndromes, such as infantile spasms or early infantile epileptic encephalopathy (EIEE), although it may also arise de novo, further complicating diagnosis and treatment [8]. The underlying etiological landscape of LGS is highly heterogeneous and encompasses genetic mutations (e.g., *SCN1A* or *STXBP1*), structural abnormalities (e.g., cortical malformations), and metabolic disorders; however, a substantial proportion of cases remain cryptogenic [5,9].

Despite its profound impact on affected individuals, most of whom experience persistent, drug-resistant seizures and severe cognitive deficits, treatment outcomes remain largely unsatisfactory [3,5,10]. The lack of a reliable biological marker, combined with the marked genetic and phenotypic heterogeneity of LGS, complicates both early diagnosis and the selection of optimal therapeutic strategies [8,11]. Current treatment approaches—including pharmacological therapies, neuromodulation, and ketogenic diets—often fail to achieve adequate seizure control or prevent cognitive decline, further exacerbating the overall disease burden on patients and caregivers [1,2]. Consequently, the quality of life is severely compromised for both patients and their families, as individuals with LGS typically face lifelong disability and limited independence [10].

Given the high degree of treatment resistance and the complex, long-term care needs associated with LGS, there is an urgent need for more effective and individualized treatment strategies. This review aims to synthesize recent advances in the understanding of LGS, spanning molecular pathogenesis, evolving diagnostic criteria, and novel therapeutic options. By adopting a “molecule-to-precision-therapy” framework, we explore emerging treatments targeting specific molecular pathways, the expanding role of genetic testing in guiding personalized care, and the potential of precision medicine to improve clinical outcomes [10,12]. Ultimately, this review seeks to provide an updated and integrated perspective on LGS that can inform future research and clinical practice, with the goal of improving treatment efficacy and quality of life for affected patients.

This article is a narrative review. We performed a focused literature search of PubMed/MEDLINE and major clinical-trial registries for English-language publications primarily from 2018 to 2025 using combinations of terms including “Lennox–Gastaut syndrome”, “developmental and epileptic encephalopathy”, “slow spike-wave”, “generalized paroxysmal fast activity”, “cannabidiol”, “ketogenic diet”, “vagus nerve stimulation”, “deep brain stimulation”, “responsive neurostimulation”, and “precision medicine”. Priority was given to randomized controlled trials, systematic reviews/meta-analyses, consensus statements, and high-quality cohort studies with direct relevance to LGS.

## 2. Electroclinical Phenotype and Disease Burden of LGS

### 2.1. Core Electroclinical Diagnostic Criteria

**Figure 1:**
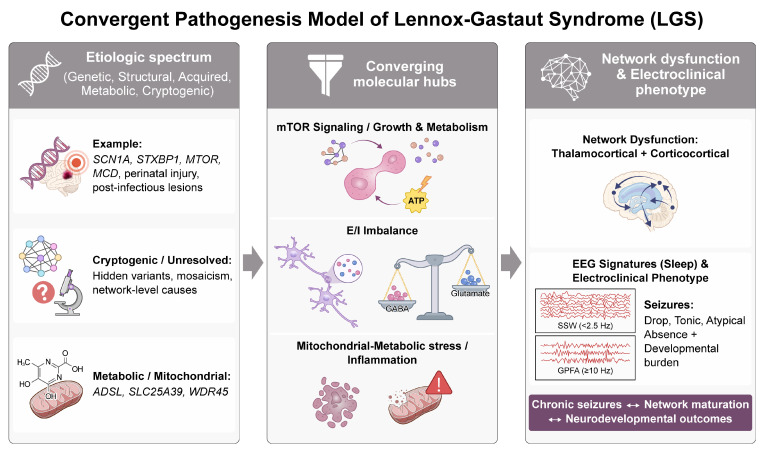
Convergent Pathogenesis Model of Lennox–Gastaut Syndrome. Abbreviations: LGS, Lennox–Gastaut syndrome; EEG, electroencephalography; E/I, excitation–inhibition; SSW, slow spike–wave; GPFA, generalized paroxysmal fast activity; mTOR, mechanistic target of rapamycin.

According to contemporary International League Against Epilepsy (ILAE) criteria, LGS is a severe DEE characterized by multiple drug-resistant seizure types beginning before 18 years of age, mandatory tonic seizures, cognitive and behavioral impairment, and a characteristic EEG pattern featuring diffuse SSW discharges and GPFA. Seizure onset typically occurs between 18 months and 8 years of age, often after infantile epileptic spasms syndrome (IESS), and most patients progress toward persistent intellectual disability and impaired adaptive functioning that extends into adulthood despite intensive treatment [5,8]. The electroclinical hallmark of LGS is a polymorphic seizure repertoire in which tonic seizures during sleep coexist with atonic (drop) attacks, atypical absence seizures, myoclonic seizures, and generalized tonic–clonic seizures, focal impaired-awareness seizures, epileptic spasms, and nonconvulsive status epilepticus. Population-based studies consistently identify tonic seizures as most frequent, followed by atypical absence, myoclonic, and atonic seizures, while stereo-EEG (SEEG) investigations link tonic and atonic events to low-voltage fast activity within distributed frontocallosal–thalamic networks [13,14]. Although focal or multifocal spikes and background slowing may reflect an underlying structural or genetic etiology, their presence does not exclude the diagnosis. These electrographic features are now incorporated into updated ILAE syndrome definitions and seizure terminology [7,14]. Collectively, the early childhood onset of multiple generalized seizure types with prominent tonic events, the mandatory EEG signature of generalized SSW with GPFA during non–rapid eye movement (NREM) sleep, and SEEG-demonstrated bilateral but stereotyped frontoparietal epileptogenic networks constitute the core operational definition of “classic” LGS. This framework enables robust differentiation from other childhood-onset DEEs with overlapping seizure and EEG phenotypes (Figure 1) [13,14,15].

### 2.2. Atypical and Overlapping Phenotypes

Beyond the canonical triad, many children who ultimately meet the diagnostic criteria for LGS follow a dynamic electroclinical trajectory, in which early DEEs—most commonly IESS—evolve toward tonic seizures and the characteristic SSW and PFA patterns [4,7,10]. Approximately half of the infants with severe DEE, and nearly 20% of childhood-onset LGS cases overall, develop following IESS, reinforcing the concept of a “syndrome in evolution” and highlighting the importance of repeated reassessment of diagnostic criteria listed in Table 1 during the follow-up [7]. This evolutionary course blurs diagnostic boundaries with other DEEs characterized by drop attacks and generalized epileptiform discharges, particularly epilepsy with myoclonic–atonic seizures (EMAtS), electrographic status epilepticus in sleep (ESES), and Dravet syndrome (DS) [2,3,4,7]. As summarized in Table 1, EMAtS shares myoclonic and atonic seizures but is distinguished by earlier onset, mandatory MAtS, generalized spike–wave activity at 2–6 Hz, and the absence of early tonic seizures. By contrast, LGS requires the presence of tonic seizures and SSW with GPFA during sleep [2,4,7]. DS and ESES may also present with multiple generalized and focal seizure types accompanied by cognitive regression comparable to that observed in LGS; however, DS is typically heralded by prolonged febrile or hemiclonic seizures during the first year of life and is frequently associated with *SCN1A* variants, whereas ESES is defined by near-continuous spike–wave activity during NREM sleep without tonic seizures [2,3,4]. Across this spectrum, shared patterns of functional disability and overlapping genetic architectures emphasize that DS, EMAtS, ESES, and LGS represent a phenotypic continuum within the broader category of DEEs. Consequently, rigorous longitudinal electroclinical phenotyping is essential for accurate classification and for anticipating long-term developmental burdens [3,16].

### 2.3. Epidemiology, Natural History, and Outcome

Epidemiologically, LGS is an uncommon yet clinically significant DEE, accounting for approximately 1–2% of all epilepsy cases and 1–10% of childhood epilepsies in population-based cohort studies. Reported annual incidence estimates range from approximately 1.9–2.1 per 100,000 children, while prevalence estimates range from 2.9 to 28 per 100,000 children for confirmed cases and increase to approximately 60.8 per 100,000 children when broader claims-based definitions are applied [10,17]. These epidemiologic findings, together with mortality rates that are more than 10-fold higher than those of the general pediatric population and substantially elevated compared with other epilepsy cohorts, underscore LGS as a prototypical lifelong DEE in which ongoing epileptic activity and underlying developmental pathology act in parallel to drive progressive disability [10,18]. In a longitudinal study of 78 adults, only 17.9% achieved seizure freedom for at least 12 consecutive months, whereas the majority experienced cumulative intellectual decline and extremely poor social outcomes. Approximately 5% of patients completed a high-school diploma, 5% married, and none attained competitive employment [17]. Consistent with these findings, a cross-sectional study of 38 adults using the Vineland Adaptive Behavior Scales, Second Edition, demonstrated universally low adaptive behavior composite scores. More than three-quarters of patients were classified as low across domains, with particularly severe impairment in daily living skills and socialization. Poorer adaptive functioning was predicted by earlier age at LGS diagnosis, persistently high seizure frequency, and more severe EEG abnormalities [1]. Across healthcare systems, administrative claims and registry-based analyses reveal substantial healthcare resource utilization among individuals with LGS, including frequent hospitalizations, emergency department visits, and the need for intensive home-based care. Estimated annual direct medical costs range from 24,000 to 80,000 USD per patient. In parallel, health-related quality of life is markedly reduced for both patients and caregivers, with greater seizure burden consistently associated with increased economic hardship and psychosocial stress [10].

## 3. Molecular and Network Pathogenesis of LGS

### 3.1. Etiologic Spectrum Underlying the LGS Phenotype

LGS arises from a broad and heterogeneous etiologic spectrum, with an identifiable cause established in approximately two-thirds of affected children [9,19]. In a contemporary pediatric cohort of 156 patients, acquired structural etiologies—predominantly perinatal hypoxic–ischemic injury and central nervous system infections such as encephalitis—accounted for 25% of cases [19]. Among nonacquired cases, structural lesions were identified in 47%, most commonly malformations of cortical development, indicating that focal cortical dysplasia and related pathologies frequently serve as substrates for the LGS epileptic network [9,13,19].

Genetic causes include monogenic epilepsies, chromosomal abnormalities, and copy-number variants, with pathogenic variants detected in approximately 30% of pediatric patients with LGS [19,20]. In a Korean cohort of 17 families with LGS or LGS-like epilepsy without structural brain abnormalities, whole-exome sequencing identified 14 pathogenic or likely pathogenic variants in genes involved in neuronal signaling or neurodevelopment, underscoring that many “nonstructural” cases are genetically determined [20]. Complementary whole-genome sequencing studies in adults with unexplained developmental and epileptic encephalopathies have further revealed pathogenic variants and tandem repeat expansions in LGS, including copy-number variants below the resolution of chromosomal microarray analysis, highlighting previously unrecognized genomic mechanisms within cryptogenic disease [21]. Beyond discrete lesions or single-gene defects, convergent network-level mechanisms are increasingly recognized. LGS is now conceptualized as a “secondary network” epilepsy, in which diverse genetic, structural, infectious, immune, metabolic, and unknown etiologies converge to disrupt shared thalamocortico and subcortical circuits [22]. Supporting this framework, studies of the endocannabinoid system in pediatric epilepsies, including LGS, have demonstrated syndrome-specific changes in cannabinoid receptor 1 (CB1) and CB2 signaling, as well as in metabolic enzymes such as fatty acid amide hydrolase and monoacylglycerol lipase. These findings suggest that dysregulated neuromodulatory and immune pathways may bridge metabolic, immune, and genetic categories of disease [23]. Nearly half of affected children in these cohorts still lack a defined etiology, yet those with unknown causes may paradoxically have better seizure outcomes than those with identified acquired or genetic etiologies, implying the existence of currently unrecognized but potentially modifiable mechanisms within the cryptogenic LGS spectrum [19].

### 3.2. Converging Molecular Pathways

Across etiologic subgroups, seizures in LGS reflect a fundamental excitation–inhibition (E/I) imbalance, in which excessive glutamatergic drive and impaired γ-aminobutyric acid (GABA)–ergic inhibition generate hyperexcitable and hypersynchronous networks at single-cell and circuit levels [24,25]. Mechanistic research shows that voltage-gated sodium, potassium, and calcium channels—together with ligand-gated N-methyl-D-aspartate (NMDA), α-amino-3-hydroxy-5-methyl-4-isoxazolepropionic acid (AMPA), and GABA_A_ receptors—collectively shape the action potential, synaptic responses, and network recruitment, thereby determining E/I balance [24,25,26]. Pathogenic variants in ion channel genes, such as *SCN1A*, *SCN2A*, *SCN8A*, *KCNQ2*, *KCNA2*, and *CACNA1A*, as well as mutations in GABA_A_ and NMDA receptor subunits, including *GABRA1*, *GABRB3*, *GRIN1*, and *GRIN2B*, are recurrently identified in DEEs, such as LGS and IESS. These findings underscore convergence on shared excitatory and inhibitory signaling pathways [24,25,27]. Accordingly, contemporary precision-medicine frameworks for LGS classify many genetic causes under channelopathies and receptor or ligand dysfunction of GABAergic and glutamatergic systems, reflecting common cellular mechanisms [26].

Convergence also occurs at the level of synaptic vesicle cycling and intracellular signaling in LGS [24,25,27,28]. *STXBP1*, which encodes the SM protein Munc18-1, regulates syntaxin-1–dependent SNARE complex assembly, vesicle docking and fusion, and neurotransmitter release. Pathogenic *STXBP1* variants cause encephalopathies, including LGS, and preferentially reduce vesicle pool size in inhibitory interneurons, thereby perturbing both glutamatergic and GABAergic transmission [24,27,28]. Similarly, genes such as *DNM1* and *IQSEC2*, which are implicated in synaptopathic DEEs and LGS phenotypes, impair endocytosis and AMPA receptor trafficking, linking presynaptic vesicle recycling and postsynaptic receptor dynamics to E/I imbalance [26,27]. In parallel, de novo variants of *MTOR* and tuberous sclerosis genes *TSC1* and *TSC2*, together with precision-therapy data, highlight mechanistic target of rapamycin (mTOR) signaling and mitochondrial respiratory chain complex I deficiency as convergent hubs in LGS. In this context, impaired energy metabolism and aberrant growth signaling exacerbate network hyperexcitability and are associated with poorer seizure and more severe EEG outcomes than non-mitochondrial forms of LGS [15,26,28].

### 3.3. Network-Level Dysfunction

Neuroimaging and electrophysiological studies converge on a distributed thalamocortical–brainstem network as the substrate underlying the SSW discharges and generalized tonic phenomena characteristic of LGS [29,30]. In combined EEG–functional magnetic resonance imaging (fMRI) studies of children with LGS, interictal epileptiform discharges activate the brainstem and centromedian/anterior thalamic nuclei. Furthermore, group-level EEG–fMRI studies, together with fluorodeoxyglucose positron emission tomography (PET) of GPFA and SSW discharges, reveal a reproducible premotor/frontoparietal cortical hotspot that is functionally connected to these thalamic hubs [29,30,31]. Experimental and computational studies of thalamocortical circuitry in generalized epilepsies demonstrate that oscillatory loops involving thalamocortical relay neurons, corticothalamic projections, and GABAergic neurons of the nucleus reticularis thalami generate spike–wave and fast rhythms, providing a mechanistic framework for the dysrhythmia expressed as SSW activity and GPFA in LGS [32]. Together with recent workshop data emphasizing LGS as a heterogeneous childhood-onset DEE, these findings support a model in which diverse etiologies converge on an immature thalamocortical–frontoparietal network, the instability of which underlies the core electroclinical phenotype [5].

Within this framework, GPFA and tonic seizures appear as closely related manifestations of a secondary network epilepsy. GPFA resembles low-voltage fast activity observed at the onset of tonic seizures and serves as a practical biomarker of network excitability in LGS [29]. EEG–fMRI and intracranial electrophysiological recordings demonstrate that GPFA engages bilateral premotor/frontoparietal cortex in conjunction with the centromedian and anterior thalamus and the brainstem, consistent with cortically driven thalamocortical and corticocortical propagation of SSW and paroxysmal fast activity [29,30,31]. In the ESTEL trial, changes in GPFA burden closely tracked seizure reduction during centromedian deep brain stimulation, establishing paroxysmal fast activity as a quantitative biomarker of thalamocortical network responsiveness [29]. More broadly, connectomics studies in drug-resistant epilepsy indicate that evolving network architecture and degrees of functional segregation influence responsiveness to surgery versus neuromodulatory interventions. These data suggest that early structural or genetic insults affecting highly connected thalamocortical and frontoparietal hubs can give rise to an LGS-like encephalopathy, in which persistent dysrhythmia manifests as SSW discharges, GPFA, and generalized tonic seizures [5,32,33].

### 3.4. From Etiology to Electroclinical Phenotype

Across the spectrum of DEEs, LGS represents a prototypical disorder in which highly heterogeneous genetic and structural etiologies converge on a relatively stereotyped pattern phenotype characterized by early-onset, treatment-resistant seizures, diffuse epileptiform activity, and progressive developmental impairment [4,34]. Large cohort and population-based studies demonstrate that pathogenic variants across numerous DEE-associated genes, copy-number variants, recessive alleles, and non-genetic insults—such as malformations of cortical development or thalamic injury—can culminate in overlapping electroclinical DEE syndromes, some of which evolve into an LGS phenotype [4,34,35]. In DEE syndromes characterized by spike–wave activation during sleep, implicated genes aggregate into two brain co-expressed modules enriched for ion channels and transcriptional regulators, illustrating how distinct loci funnel into shared molecular nodes that are relevant to generalized spike–wave and sleep-activated epileptiform discharges [34]. Mechanistic reviews of DEEs further emphasize that many of these genes participate in common pathways controlling neuronal excitability, synaptic transmission, neurodevelopment, and bioenergetics, despite wide variation in their primary functions [4,35,36].

Collectively, these data support a conceptual model in which diverse etiologies converge on a limited set of cellular and network-level pathways—particularly those governing E/I balance, synaptic plasticity, and thalamocortical synchrony—that shape the characteristic electroclinical phenotype of LGS [4,34,35,36]. Within this framework, patients can be stratified not only by the proximal cause—such as a single-gene variant, multigene context, copy-number variant, or structural lesion—but also by the dominant downstream pathway or network module that is perturbed [4,34,36,37]. High-yield genomic technologies, including whole-exome and whole-genome sequencing, now explain the etiology in approximately half of individuals with DEEs, enabling classification into mechanistic categories such as channelopathies, synaptopathies, and metabolic and transcriptional disorders that are directly relevant to LGS pathobiology [4,34,35,36]. Linking these etiologic categories to systems genetics analyses of gene co-expression and molecular networks informs therapeutic strategies that span gene- or variant-specific precision therapies, pathway-directed pharmacologic modulation, and neuromodulatory approaches targeting shared thalamocortical circuits [34,35,36]. In LGS and related DEEs, such a convergence model provides a rational foundation for precision trial design, allowing interventions to be tailored to both the etiologic tier and the network node at which disparate disease mechanisms intersect [34,35]. These heterogeneous etiologic and molecular mechanisms ultimately converge on a shared network phenotype, as summarized in Figure 1.

Figure 1 illustrates a convergent pathogenesis model of LGS, highlighting how heterogeneous etiologies funnel into shared molecular and network-level dysfunction to generate the characteristic electroclinical phenotype. Diverse upstream causes—including genetic variants, structural brain abnormalities, acquired insults, metabolic and mitochondrial disorders, and unresolved or cryptogenic mechanisms—converge on common molecular hubs such as excitation–inhibition (E/I) imbalance, dysregulated synaptic transmission, altered growth and metabolic signaling (including mTOR-related pathways), and bioenergetic stress. These convergent mechanisms disrupt thalamocortical and corticocortical networks, leading to network instability that manifests as hallmark electroencephalographic features, including generalized slow spike–wave discharges (≤2.5 Hz) and generalized paroxysmal fast activity (≥10 Hz), particularly during non-rapid eye movement sleep. At the clinical level, this network dysfunction underlies the core electroclinical phenotype of LGS, characterized by drop seizures, tonic seizures, atypical absence seizures, and a high burden of developmental and cognitive impairment. The bidirectional interaction between chronic epileptic activity, aberrant network maturation, and adverse neurodevelopmental outcomes further emphasizes LGS as a network-driven developmental and epileptic encephalopathy rather than a disorder defined by a single molecular defect.

## 4. Precision Diagnostic Workflow for LGS

### 4.1. When to Suspect LGS in Clinical Practice

LGS should be suspected in children who present with multiple seizure types, particularly tonic seizures, atypical absence seizures, and atonic drop attacks, which are characteristic features of the disorder [7]. These seizures typically emerge between 3 and 5 years of age and are often preceded by a history of infantile spasms or focal seizures [4]. Clinicians should maintain a high index of suspicion in children with treatment-resistant epilepsy accompanied by developmental delays and behavioral regression [34]. A thorough evaluation of seizure semiology, in conjunction with EEG evaluation, is essential to confirm the diagnosis [38]. A hallmark EEG finding in LGS is the presence of generalized SSW discharges, most prominently observed during sleep [39].

The evolution from infantile spasms or focal seizures to a more generalized LGS pattern is often gradual and marked by the development of other seizure types over time [4]. It is crucial to recognize that not all children with LGS present with the same symptoms initially, and early identification can therefore be challenging [39]. A history of developmental regression, combined with the appearance of tonic or atonic seizures, should prompt further diagnostic investigation, including EEG monitoring and neuroimaging [38]. This progression underscores the importance of early detection and personalized treatment plans aimed at controlling seizures and mitigating associated cognitive and behavioral impairments [34].

### 4.2. EEG as the Diagnostic Anchor

EEG plays a central role in the diagnosis of LGS. The minimum electrographic criteria for LGS include the presence of generalized SSW discharges, particularly during NREM sleep, and GPFA during wakefulness [34]. However, these findings may evolve with disease progression, especially in adult patients, complicating early detection. Furthermore, EEG patterns can be altered by anticonvulsant medications, potentially leading to misinterpretation, particularly in the presence of subtle spike–wave activity or background slowing [40]. In cases where the clinical diagnosis remains uncertain, prolonged video-EEG monitoring, including sleep recording, is strongly recommended, especially for adult patients transitioning from pediatric care [41]. Sleep-associated changes in spike–wave activity, such as the appearance of generalized spikes and polyspike–wave discharges, provide important diagnostic clues for LGS [42]. High-density EEG offers superior resolution and enhances the detection of focal or multifocal discharges that may not be evident on conventional EEG recordings. These advanced techniques provide valuable information regarding seizure semiology and brain network function, thereby enabling more accurate diagnosis and precise monitoring of treatment responses [40,42].

### 4.3. Neuroimaging and Genetic Work-Up

MRI remains a cornerstone in the diagnosis of LGS. Standard protocols incorporating T1-weighted, T2-weighted, and fluid-attenuated inversion recovery sequences provide vital information regarding structural brain abnormalities, including cortical malformations such as polymicrogyria, which are frequently identified in patients with LGS [43]. Additionally, advanced neuroimaging techniques such as fMRI and PET offer valuable insights into assessing functional connectivity in the brain. These techniques help identify disrupted neural networks involved in seizure generation and propagation [39,44]. PET, for example, is useful in detecting metabolic abnormalities and areas of brain dysfunction that may not be evident on standard MRI [34].

Genetic testing has become an integral component of the diagnostic workflow for LGS, especially in cases in which structural imaging does not reveal a clear etiology. Whole-exome and whole-genome sequencing have shown high diagnostic yields in patients with DEEs such as LGS [43]. Targeted gene panels, along with copy-number variation analysis, can also reveal pathogenic variants in key genes, such as *GRIN2A*, *SCN1A*, and *KCNQ3*, which are frequently implicated in LGS [39,43,45]. Pathogenic variants identified through these genetic panels, particularly in genes such as *KCNQ2* and *STXBP1*, have been associated with LGS and other related DEEs. These genetic findings are crucial for confirming the diagnosis of LGS and guiding targeted treatments, such as the use of ketogenic diets and sodium channel blockers, which have demonstrated effectiveness in managing seizures associated with these variants [46]. Copy-number variation analysis is particularly valuable for detecting chromosomal deletions or duplications that contribute to the LGS phenotype [34,39]. When combined with clinical features and neuroimaging findings, genetic data support the development of targeted treatment strategies for LGS. Table 2 summarizes the key genes associated with LGS and their respective variants, providing valuable insights into the genetic underpinnings of the syndrome.

Table 2 highlights representative genes associated with LGS and emphasizes their clinical relevance to the characteristic electroclinical phenotype rather than simple gene–phenotype associations.

## 5. Current Treatment Strategies for LGS

### 5.1. Broad-Spectrum and First-Line Anti-Seizure Medications (ASMs)

Broad-spectrum ASMs remain the cornerstone of pharmacologic treatment for LGS, given the coexistence of multiple generalized and focal seizure types and the near-universal need for polytherapy [67,68]. Valproate is most consistently recommended as a first-line agent in expert consensus statements and treatment algorithms, despite the absence of randomized controlled trials specific to LGS [68]. Extensive clinical experience, availability in multiple formulations, and a broad-spectrum mechanism of action targeting GABAergic tone and modulation of sodium channels explain its central role in initial treatment strategies [67,68]. However, valproate use requires careful risk–benefit assessment because of age-dependent hepatotoxicity, teratogenicity potential, and metabolic adverse effects, particularly in young children and females of childbearing potential [67,68].

Given that seizure freedom is rarely achieved with monotherapy, rational polytherapy is a core principle of LGS management. This approach prioritizes combinations of ASMs with complementary mechanisms of action while minimizing pharmacokinetic and pharmacodynamic toxicity [68,69]. Well-established synergistic combinations include valproate combined with lamotrigine and cannabidiol combined with clobazam, both of which exhibit enhanced efficacy but require close monitoring for dose-dependent adverse effects [68,69]. Clinically significant drug–drug interactions are common in LGS owing to polypharmacy. Valproate increases serum concentrations of lamotrigine and rufinamide and may precipitate hyperammonemia when co-administered with topiramate [67,69]. Cannabidiol markedly elevates levels of N-desmethylclobazam, thereby increasing the risk of sedation, and cenobamate can dramatically amplify this interaction when added to clobazam-based regimens [67,69]. Accordingly, rational polytherapy in LGS requires iterative reassessment, dose adjustment guided by clinical response and tolerability, and avoidance of excessive ASM layering that increases adverse-effect burden without meaningful seizure benefits [67,68,69]. These considerations underscore that first-line and broad-spectrum ASMs in LGS are selected primarily based on mechanistic complementarity and network-level coverage rather than expectations of seizure freedom. In selected cases, molecular information may further inform pharmacologic choices, particularly when variants implicate pathways or targets known to influence antiseizure medication response or tolerability.

### 5.2. LGS-Focused and Newly Approved Agents

Lamotrigine, clobazam, rufinamide, topiramate, and felbamate constitute the core group of LGS-focused ASMs supported by pivotal randomized or controlled studies, with efficacy primarily evaluated against drop seizures and overall seizure burden [68,70,71]. Among these agents, clobazam consistently demonstrates strong efficacy in reducing drop attacks, while rufinamide and lamotrigine show broader activity across tonic, atonic, and atypical absence seizures, supporting their frequent use as syndrome-specific options [68,70]. Topiramate and felbamate provide additional benefit in selected patients, particularly for tonic and generalized tonic–clonic seizures; however, their use is often limited by cognitive or systemic adverse effects, necessitating careful balancing of seizure reduction against tolerability [68,70].

Cannabidiol has emerged as a robust, evidence-based adjunctive therapy for LGS, supported by class I randomized controlled trials demonstrating clinically meaningful reductions in drop seizures at doses of 10–20 mg/kg/day [12,68]. In these studies, cannabidiol reduced drop-seizure frequency by approximately 37–42% compared with placebo and also improved non-drop seizure types, confirming efficacy across the heterogeneous seizure spectrum of LGS [12,68,72]. Subsequent post hoc and real-world analyses have reported increases in seizure-free days and caregiver-reported global improvement, emphasizing benefits that extend beyond conventional responder thresholds [68,73]. Mechanistically, cannabidiol acts via multi-target, CB1/CB2-independent pathways, including GPR55 antagonism, transient receptor potential–channel modulation, and adenosine-mediated dampening of network excitability, providing a biological rationale for its broad efficacy in LGS [73,74]. Consensus recommendations emphasize interaction-aware titration, routine monitoring of liver function—especially in patients receiving concomitant valproate—and proactive management of clobazam-related sedation to optimize long-term outcomes [72,73]. Adverse events, drug–drug interactions, and regulatory approval status vary across regions and should be considered when translating trial results into routine clinical practice. Importantly, individual response is heterogeneous, and short-term trial endpoints may not fully capture long-term tolerability or sustained effectiveness in routine care. In particular, clinically meaningful drug–drug interactions (notably with clobazam) and treatment-limiting adverse effects should be considered when interpreting real-world persistence and benefit [72,73].

Beyond established therapies, cenobamate has gained increasing attention as a promising option for highly refractory LGS, based on accumulating real-world evidence and recent systematic analyses across DEEs [70,71,75]. In a meta-analysis comprising predominantly LGS and LGS-overlapping DEE cohorts, cenobamate achieved pooled ≥50% seizure-reduction rates of approximately 57% and seizure freedom in about 10% of patients, despite extensive prior treatment failures and prolonged disease duration [75]. Notably, LGS-specific case series within this analysis reported responder rates ranging from approximately 38% to 75%, with seizure-free outcomes observed in a meaningful subset, exceeding expectations for many newer adjunctive therapies used in comparable populations [71,75]. Although treatment-emergent adverse events such as somnolence, dizziness, and ataxia were common, they were generally manageable with slow titration and adjustment of concomitant ASMs. Overall retention rates of approximately 85–90% in observational cohorts underscore both the effectiveness and tolerability of cenobamate in carefully selected patients with LGS [75,76]. However, current data are largely derived from real-world and observational studies, and long-term safety and controlled trial data specific to LGS remain limited.

## 6. Non-Pharmacologic, Network-Targeted, and Surgical Therapies

### 6.1. Ketogenic and Related Dietary Therapies

Ketogenic and related dietary therapies are well-established non-pharmacologic options for LGS, particularly in drug-resistant cases characterized by multiple coexisting seizure types [77,78]. The classical ketogenic diet has the longest evidence base, while less restrictive variants—including the medium-chain triglyceride diet, modified Atkins diet (MAD), and low-glycemic-index treatment (LGIT)—have been developed to improve tolerability and long-term adherence [77,79]. Across randomized and observational studies in drug-resistant epilepsy, dietary therapies consistently achieve a ≥50% reduction in seizure frequency in a substantial proportion of patients, with LGS cohorts showing meaningful responses across tonic, atonic, and atypical absence seizures [77,78]. Accordingly, these approaches are increasingly regarded as core network-targeted therapies rather than adjunctive lifestyle measures [79,80].

Multiple complementary mechanisms have been proposed to explain the anti-seizure effects of ketogenic therapies, extending beyond the induction of ketosis alone [77,78]. Metabolic shifts from glucose to ketone utilization enhance mitochondrial bioenergetics and reduce oxidative stress, processes that are particularly relevant in DEEs [77,81]. In addition, ketone bodies modulate neurotransmitter balance by increasing GABAergic tone and reducing glutamatergic excitability, while also influencing ion channel function and adenosine signaling at the network level [77,79]. Anti-inflammatory effects, including reductions in pro-inflammatory cytokine levels, along with emerging evidence on gut microbiome modulation, further support a systems-level impact on epileptic networks rather than isolated seizure generators [79,81].

Evidence specific to LGS indicates that dietary therapies can produce clinically meaningful seizure reduction and, in some cases, seizure freedom, with responder rates often comparable to or exceeding those of additional ASMs [77,80]. Practical considerations strongly influence outcomes, including age at initiation, method of nutritional delivery, and family capacity for adherence, with MAD and LGIT often favored in adolescents or patients receiving enteral feeding owing to their greater flexibility [77,79]. Long-term safety profiles are generally acceptable when dietary therapies are implemented under specialist supervision; however, metabolic contraindications and the risk of nutritional deficiencies require careful screening and monitoring [78,79]. Within multimodal treatment algorithms for LGS, ketogenic and related dietary therapies therefore represent a rational, network-directed intervention that complements pharmacologic and surgical strategies [79,80,82].

### 6.2. Neuromodulation

Vagus nerve stimulation (VNS) is thought to exert its anti-seizure effects by desynchronizing abnormal neuronal activity and modulating complex distributed circuits between the brainstem and various cortical structures [83,84]. The procedure involves implantation of a stimulator that targets the left vagus nerve, which projects directly to the nucleus tractus solitarius to influence diffuse cortico-subcortical networks [85]. Long-term real-world data from the CORE-VNS study demonstrate clinically meaningful efficacy, with responder rates (≥50% seizure reduction) of 66.7% for focal seizures and 47.4% for generalized seizures after 24 months of treatment. Approximately 20% of patients with LGS in this cohort achieved an 80% or greater reduction in overall seizure frequency [85]. Beyond seizure reduction, VNS has been associated with secondary benefits such as increased alertness, improved quality of life, and reduced postictal severity. Adverse effects such as coughing or hoarseness can occur during the stimulation phase but are typically mild, transient, and tend to diminish as patients acclimate to the device [86,87].

Deep brain stimulation (DBS) has emerged as a vital circuit-level intervention for LGS, with the centromedian nucleus of the thalamus serving as the preferred target owing to its extensive connectivity across the brain [83,87]. By delivering electrical pulses to this nucleus, DBS modulates the diffuse bilateral networks implicated in LGS and interferes with the thalamocortical recruitment that facilitates seizure spread. [79,83,88]. Newer DBS devices feature advanced chronic sensing of local field potentials and the use of directional leads, allowing clinicians to refine therapy based on the patient’s specific brain activity patterns. This precision approach helps minimize potential side effects such as transient drowsiness or stimulation-related paresthesia. Accurate electrode placement is crucial for therapeutic success and requires specialized, high-resolution neuroimaging to clearly visualize the thalamic targets [83,86].

Responsive neurostimulation (RNS) utilizes an adaptive, closed-loop system that provides continuous and near-real-time monitoring and delivers on-demand stimulation only in response to detected abnormal patterns [83,86]. This on-demand, localized desynchronization at the source is particularly beneficial when seizure foci involve the eloquent cortex, and its adaptive nature often confers longer battery life compared with continuous systems [86,87]. In LGS, RNS can be implemented using a corticothalamic approach, with leads placed in both the thalamic nuclei and specific cortical areas. Recent clinical reports suggest that combining neuromodulation techniques—such as adding DBS or RNS in patients with optimized VNS therapy—may yield additional, synergistic seizure reduction [83,86,87]. Management strategies incorporate objective electrophysiologic biomarkers, such as GPFA, to measure real-time response and accelerate treatment titration. These device-based approaches can be interpreted within the thalamocortical network model of LGS, in which stimulation aims to modulate distributed cortico–subcortical circuits and measurable electrophysiologic markers (e.g., GPFA burden) that track network excitability and treatment response [29,31]. Ultimately, because no single device is universally superior, the choice of neuromodulation strategy depends on individual seizure types, anatomical suitability, and the specific goals of the patient, family, and surgical team [79,86].

### 6.3. Epilepsy Surgery

Resective surgery is the primary treatment of choice when a focal epileptogenic zone can be identified through comprehensive diagnostic evaluation [79,89]. Although LGS has traditionally been viewed as a generalized epilepsy, careful presurgical assessment using 3T MRI, PET, and single-photon emission computed tomography often reveals resectable focal abnormalities, such as malformations of cortical development or unilateral destructive lesions [89,90]. Procedures involving hemispherectomy, unilobar resection, and multilobar resection can yield substantial and often immediate seizure reduction, with hemispherectomy achieving the highest seizure-free rates of approximately 71.4% [79,80,90]. Favorable outcomes are strongly associated with concordant findings across multimodal imaging and EEG, underscoring the clinical importance of identifying focal features even when preoperative scalp EEG shows generalized patterns [89,90]. These curative approaches target the primary foci driving abnormal networks, often resulting in significant improvements in baseline EEG activity and overall neurodevelopmental progress [79,80,90].

When resective surgery is not feasible because of multifocal or unknown seizure onset, palliative disconnection procedures such as corpus callosotomy (CC) represent a critical component of symptom management [89,91]. CC disconnects the cerebral hemispheres to attenuate interhemispheric synchronization, which is particularly effective in reducing disabling drop attacks and injurious tonic or atonic seizures [89,91,92]. Although anterior callosotomy is often favored to minimize the risk of disconnection syndrome, complete CC has been associated with greater overall seizure reduction in several studies [80,91]. Recent advancements in minimally invasive techniques, including stereotactic laser anterior CC and laser interstitial thermal therapy, have demonstrated efficacy comparable to that of traditional open craniotomy, while offering shorter hospital stays and reduced complication rates [79,89]. Furthermore, CC offers significant benefits for children who have previously failed VNS, with approximately 83% of these patients achieving at least a 50% reduction in drop attacks [91,92].

The timing of surgical intervention is a critical determinant of outcome. Shorter intervals between seizure onset and surgery are strongly associated with a higher likelihood of achieving seizure freedom and more favorable neurocognitive outcomes [80,89,90]. Despite this evidence, delays in surgical evaluation remain a major barrier, with many patients undergoing prolonged courses of ineffective pharmacological management after failing multiple ASMs [89,92]. Early surgical intervention targets the “secondary network” pathophysiology of LGS before epileptic circuits become irreversibly destabilized and prevents the progressive cognitive decline and behavioral regression associated with chronic seizure activity [89,90]. Clinical evidence indicates that patients who undergo resective procedures within 5 years of seizure onset are significantly more likely to remain seizure-free than those treated a decade or more after onset [79,90]. Consequently, updated management protocols advocate for surgical consideration earlier in the disease course to optimize long-term social quotients and decrease the risk of mortality [80,89,90,92].

## 7. From Molecular Insights to Precision, Targeted, and Multimodal Therapeutic Strategies

### 7.1. Etiology- and Pathway-Directed Precision Therapies: What Is Actionable Now, and What Is Emerging

In LGS, “precision therapy” is most feasible when it is anchored to (i) a defined etiology (e.g., structural, genetic, or metabolic) and/or (ii) a dominant downstream pathogenic pathway (e.g., mTOR hyperactivation or channel/receptor dysfunction), rather than to the LGS electroclinical label alone. This is because multiple upstream causes converge on shared mechanisms of neuronal excitability and thalamocortical network dysfunction that generate the LGS phenotype [4,5,22,26]. Accordingly, a practical framework is to prioritize established disease- or pathway-directed interventions when available and then layer mechanism-informed symptomatic therapies—including ASMs, diet, neuromodulation, and surgery—around that core strategy [8,22,26,93].

Actionable “cause-specific” strategies already exist for several clinically important molecular subgroups that can present as, or evolve into, LGS. mTOR-pathway epilepsies—including TSC and related mTORopathies—represent prototypical examples, in which mTOR inhibition can be conceptualized as disease-modifying therapy rather than purely symptomatic seizure suppression [26,52,93]. Similarly, metabolic epilepsies in the LGS spectrum justify early deployment of targeted metabolic interventions: the ketogenic diet serves a paradigmatic precision intervention for glucose transporter type 1 deficiency and may also be rational in other genetic/metabolic contexts where bioenergetic support is relevant [77,79,81,93]. In mitochondrial-associated LGS phenotypes, “precision” often entails risk-aware drug selection combined with supportive metabolic strategies, given evidence that mitochondrial respiratory chain dysfunction can influence seizure burden and EEG outcomes in LGS [15]. The key point for clinical implementation is that the therapeutic leverage is greatest when the upstream driver is identified sufficiently early to influence the treatment trajectory [8,22,26,93].

A second layer of precision is “mechanism-informed” selection—or avoidance—of existing therapies based on the functional consequences of genetic variation. Channelopathies and receptoropathies are common mechanistic categories across DEEs that can produce an LGS phenotype [25,26]. For example, sodium-channel gain-of-function disorders (classically *SCN2A*/*SCN8A* early-onset gain-of-function variants) may respond to sodium channel blockers, whereas sodium-channel loss-of-function disorders that impair inhibitory interneurons (e.g., *SCN1A* loss-of-function variants) often warrant avoidance of sodium channel–blocking strategies [25,26,93]. Analogously, disorders of glutamatergic NMDA receptors (GRIN-related) and GABA receptors (GABA_A_ subunit genes) support a function-first approach, in which antagonists, modulators, or GABAergic strategies are selected depending on gain- versus loss-of-function effects. However, real-world availability and trial-level evidence remain uneven across genes and variants [26,93]. For synaptic-vesicle and synaptopathic DEEs that can culminate in LGS (e.g., *STXBP1*-related disorders), emerging mechanism-based approaches—including chemical chaperone concepts—are increasingly discussed as translational candidates, although they remain investigational for routine LGS care [26,28,93]. Because variant interpretation is not always straightforward, this tier of precision is most robust when paired with functional annotation, multidisciplinary care review, and careful outcome monitoring [26,93].

A third, rapidly evolving tier is gene- and transcript-targeted therapy development across DEEs, which will inevitably encompass subsets of patients currently diagnosed clinically as having LGS. Current DEE pipelines now explicitly include antisense oligonucleotides and gene-therapy strategies for selected channelopathies, and small-molecule or pathway-modifying strategies for receptor and synaptic disorders. Examples include modulators such as gaboxadol, radiprodil, and L-serine for receptor-related mechanisms, along with pathway-directed approaches targeting synaptic signaling disorders [93]. For LGS, the near-term implication is not the imminent availability of an “LGS-specific gene therapy,” but rather the need for LGS clinics to be structured to (i) identify eligible molecular subgroups via high-yield genetic testing, (ii) contribute to natural history studies and biomarker development, and (iii) maintain trial readiness for mechanism-defined interventions [22,26,93]. Recent DEE pipelines increasingly include gene- and transcript-targeted programs (e.g., antisense oligonucleotides, gene replacement, and mechanism-directed small molecules) that are not “LGS-specific” but may apply to molecular subgroups that present as, or evolve into, an LGS phenotype. In practice, the near-term clinical value of these programs is to enhance trial readiness: identifying eligible molecular subgroups through high-yield testing, aligning families with realistic expectations for investigational therapies, and contributing to natural history and biomarker efforts. At present, most such approaches remain experimental for routine LGS care, and their applicability is constrained by variant heterogeneity, developmental timing, and limited long-term outcome data [93]. This “precision infrastructure” becomes a therapeutic strategy, as it determines which patients can access emerging disease-modifying approaches as they become viable [93].

### 7.2. Operationalizing Precision Care in LGS: Seizure-Type and Network-Guided Multimodal Sequencing with Meaningful Outcomes

**Figure 2:**
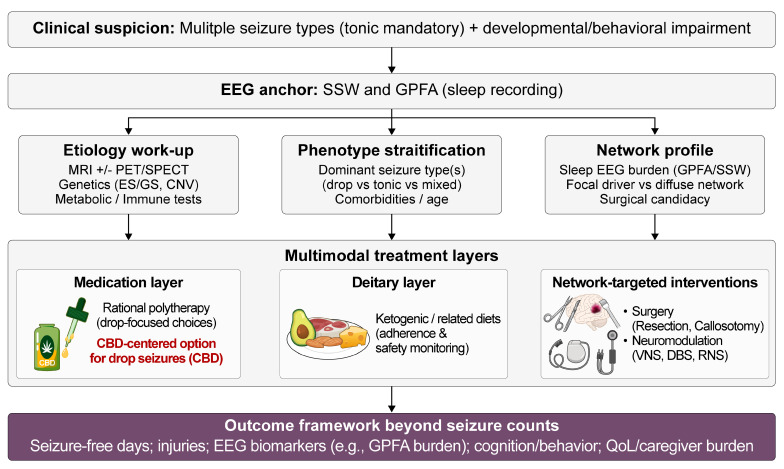
Precision Diagnostic and Multimodal Treatment Pathway for Lennox–Gastaut Syndrome. Abbreviations: LGS, Lennox–Gastaut syndrome; EEG, electroencephalography; SSW, slow spike–wave; GPFA, generalized paroxysmal fast activity; CBD, cannabidiol; VNS, vagus nerve stimulation; DBS, deep brain stimulation; RNS, responsive neurostimulation; QoL, quality of life.

In routine clinical management of LGS, the most immediately actionable form of “precision” is often phenotype-first. For patients without an identifiable molecular driver—still a substantial proportion of LGS—this framework should be operationalized primarily as phenotype- and network-guided sequencing with realistic expectations, rather than as gene-specific therapy [8,22,26]. This approach aligns treatment choices with (i) the dominant disabling seizure type(s)—especially drop seizures and tonic seizures; (ii) the suspected network organization, such as focal driver versus diffuse thalamocortical involvement; and (iii) patient-specific constraints, such as age, comorbidities, and feasibility of devices/surgery/diet [8,22,68,94]. This framework is particularly relevant because many LGS trials were powered using “drop seizures” as endpoints, whereas families and clinicians must manage multiple seizure types with different injury risks and response profiles [68,94]. A precision-oriented diagnostic and treatment framework integrating etiology, seizure phenotype, and network characteristics is outlined in Figure 2.

Figure 2 presents a precision-oriented diagnostic and multimodal treatment pathway for LGS, integrating electroclinical assessment, etiologic evaluation, and network-guided therapeutic decision-making. Following clinical suspicion based on multiple seizure types with mandatory tonic seizures and developmental impairment, EEG serves as the diagnostic anchor through identification of slow spike–wave discharges and generalized paroxysmal fast activity, particularly during sleep. Patients are subsequently stratified along three complementary axes: underlying etiology (genetic, structural, metabolic, or acquired), dominant seizure phenotype with particular emphasis on drop seizures, and network profile derived from EEG burden and neuroimaging findings, including the presence of focal drivers versus diffuse thalamocortical involvement. This stratification informs a layered treatment strategy encompassing rational polytherapy, dietary interventions, and network-targeted procedures. Within the pharmacologic layer, cannabidiol is highlighted as a central option for drop seizures, reflecting its consistent evidence base and clinically meaningful benefits. Surgical and neuromodulatory approaches, including corpus callosotomy, vagus nerve stimulation, deep brain stimulation, and responsive neurostimulation, are positioned as adjunctive network-level interventions for selected patients. Treatment outcomes extend beyond seizure counts to include seizure-free days, injury reduction, electrographic biomarkers, cognitive and behavioral function, and caregiver-relevant quality-of-life measures, underscoring a comprehensive precision-care framework for LGS.

Recent syntheses focusing explicitly on seizure-type–specific responses highlight several pragmatic patterns that can refine “precision” even when etiology remains unknown. Across available evidence, clobazam demonstrates particularly strong effects on drop seizures, whereas rufinamide appears enriched for atonic (drop) seizure benefit relative to tonic seizures, and lamotrigine/topiramate shows broader—but variable—coverage across tonic/atonic/atypical absence/myoclonic seizures depending on the patient context and study design [68,70,94]. Cannabidiol has robust evidence for reducing drop seizures and also demonstrates clinically relevant effects on non-drop seizures, including tonic and convulsive seizure categories in post hoc analyses, supporting its use when the seizure burden extends beyond drop attacks [12,72,94]. Fenfluramine is also discussed in the context of seizure-type responsiveness, notably generalized tonic–clonic seizures in available syntheses, although the practical role in LGS still requires careful individualized positioning within complex polytherapy regimens and adverse-effect constraints [67,93]. Importantly, the seizure type–specific literature also highlights a key limitation: many datasets were not designed or powered to demonstrate differential effects across individual seizure types. Accordingly, seizure-type guidance should be interpreted as “probability-shifting” rather than deterministic [94].

A complementary axis of precision involves network-guided selection of neuromodulation and surgical interventions, and these strategies are increasingly framed around matching interventions to the presumed dominant circuit dysfunction rather than to seizure counts alone [31,83,94]. Conceptual mappings linking common seizure types to large-scale networks suggest plausible “best-fit” strategies: tonic seizures, associated with frontal–thalamic and brainstem-linked circuits, may be particularly amenable to thalamic/circuit neuromodulation; atonic and myoclonic seizures, implicating motor network, often respond well to disconnection strategies such as CC; and focal-onset features involving temporal–limbic circuits identify patients most likely to benefit from resection and/or responsive neurostimulation when appropriate selection criteria are met [89,94]. Although simplified, this network lens helps translate heterogeneous electroclinical presentations into a coherent multimodal plan, especially when combined with modern imaging/genetic evaluation and prolonged EEG characterization [31,83].

Recent comparative neuromodulation meta-analyses provide a quantitative anchor for counseling and strategy selection across devices. Across 54 studies involving 1350 patients with LGS, the pooled ≥ 50% responder rate with neuromodulation was 55.4%, with higher pooled responder rates reported for DBS (69.7%) and RNS (63.0%) than for VNS (50.6%). Intervention-type moderated response and later epilepsy onset predicted better response within DBS cohorts [95]. Quality-of-life improvements were frequently described (notably improved alertness/attention), although adverse-event profiles differed: stimulation-related side effects were more typical with VNS, whereas DBS and RNS demonstrated higher rates of serious device-related issues in the available literature [95]. Practically, these data support a precision-oriented discussion focused not only on whether a device is effective, but on which device best aligns with the patient’s network phenotype, age, surgical candidacy, and family priorities—consistent with expert guidance emphasizing individualized target selection, programming, and multimodal integration rather than a one-device-fits-all approach [83,86,95].

Real-world practice patterns in pediatric LGS surgery further underscore why “precision care” must be feasible and system-aware, not solely biologically rational. In a cross-sectional survey of 32 pediatric epilepsy centers, most institutions considered surgery after the failure of three to four ASMs. All centers offered VNS and CC, and the majority offered resection or hemispherectomy and RNS, whereas DBS and laser ablation were less uniformly available [96]. Decision-making was strongly influenced by seizure frequency, family preferences, and multidisciplinary input. Etiology and MRI findings were particularly critical for resection/hemispherectomy decisions, and there were scenario-specific trends such as a preference for CC when drop seizures predominate and less frequent DBS/RNS consideration in children under 5 years of age [96]. These findings are clinically important because they reveal where “ideal” precision pathways may be constrained by access, age-related factors, insurance/financial barriers, or the need for future additional diagnostic evaluation. They also highlight opportunities where consensus pathways and earlier referral to comprehensive centers can create the largest real-world gains [89,92,96].

Finally, a precision strategy is only as strong as the outcomes used to define success. For LGS, measures that capture “time without seizures” may reflect family experience more faithfully than percentage reduction alone. A post hoc analysis of two randomized controlled trials of cannabidiol introduced seizure-free days as a clinically meaningful endpoint. Over a 14-week treatment period, least-squares mean increases in drop seizure–free days per 28 days were 2.81 with placebo versus 5.64 with cannabidiol 10 mg/kg/day (CBD10) and 6.45 with cannabidiol 20 mg/kg/day (CBD20), corresponding to least-squares mean differences versus placebo of 2.83 (CBD10) and 3.64 (CBD20), respectively. The total number of seizure-free days also improved with least-square mean differences versus placebo of 2.63 (CBD10) and 3.50 (CBD20), respectively [97]. In parallel, objective network biomarkers are becoming increasingly actionable in device-based therapies. Paroxysmal fast activity has been proposed as a measurable marker of treatment response in thalamic DBS for LGS, linking electrophysiologic network change to clinical improvement and providing a potential bridge between mechanism and outcome [29]. Taken together, seizure-free days and network biomarkers—alongside neurodevelopmental, behavioral, and quality-of-life measures—strengthen precision care by enabling more individualized “go/no-go” decisions when sequencing medications, diets, devices, and surgery, especially in a syndrome where complete seizure freedom is uncommon and comorbidity burden is high [10,82,95,97].

## 8. Discussion

Accumulating molecular and network-level evidence supports LGS as a convergent DEE in which heterogeneous genetic, structural, and acquired etiologies disturb shared thalamocortical networks rather than producing syndrome-specific mechanisms [98]. This framework explains both the marked treatment resistance characteristic of LGS and the observation that the most effective therapies tend to modulate network excitability rather than target a single molecular defect. Within this context, cannabidiol has emerged as one of the most consistently effective pharmacological therapies for drop seizures, a defining and highly morbid seizure type in LGS. Comparative evidence synthesis shows that cannabidiol ranks among the most effective agents for reducing drop seizures, with effects that are robust across clinical trials and extend beyond seizure counts to outcomes such as seizure-free days, which more accurately reflect daily functioning and caregiver burden [99].

Despite substantial advances in the understanding and management of LGS, current evidence remains limited by heterogeneous study populations, reliance on observational data, and a relative scarcity of syndrome-specific randomized controlled trials, underscoring the need for prospective and mechanistically stratified studies. Although neuromodulation strategies demonstrate moderate-to-high responder rates, the majority of available data are nonrandomized and heterogeneous, limiting definitive conclusions regarding comparative effectiveness and long-term safety [95]. Conversely, emerging pathway-directed agents highlight the difficulty of translating molecular targets into clinically meaningful benefit. For example, pooled randomized data indicate that soticlestat does not provide a clear overall responder advantage in LGS and is associated with an increased incidence of adverse events, despite signals in selected treatment phases [100]. Substantial gaps in knowledge persist, including limited data on under-represented etiologies, age-dependent treatment effects, and long-term cognitive and quality-of-life outcomes. These limitations underscore the need for a more integrated and clinically grounded precision framework.

## 9. Conclusions and Future Directions

LGS remains a severe, network-driven DEE in which complete seizure freedom is uncommon. Current evidence supports a pragmatic, multimodal approach grounded in early etiologic evaluation and realistic goals. Among available therapies, cannabidiol shows the most consistent and clinically meaningful efficacy for reducing drop seizures and overall seizure burden, with outcomes aligning closely with patient- and caregiver-relevant measures. Neuromodulation and surgical strategies provide additional network-level options for selected patients but require careful risk–benefit assessment. Future research should prioritize etiology- and pathway-stratified clinical trials, define optimal multimodal sequencing, and incorporate real-world data alongside neurodevelopmental outcomes to advance precision care for individuals with LGS.

## Figures and Tables

**Table 1:** Core electroclinical features of LGS and key differential diagnoses.

Syndrome	Seizure Pattern	EEG Hallmarks	Age at Onset/Course	Distinguishing Criteria vs. LGS
LGS (DEE-SSW)	Mandatory tonic seizures plus ≥1 additional type: atypical absence seizures, atonic (drop) seizures, myoclonic seizures, focal impaired awareness, generalized tonic–clonic seizures, epileptic spasms, and frequent nonconvulsive status epilepticus.	Generalized SSW with frontal predominance, in runs. Commonly characterized by GPFA during slow-wave sleep, abnormal background with theta–delta slowing, and focal/multifocal spikes.	Typical occurrence at 18 months–8 years of age (peak 3–5 years). Continues into adulthood with drug-resistant seizures and moderate–severe intellectual disability.	Canonical triad of tonic-predominant multiple seizure types, SSW + GPFA, and developmental impairment; falls from tonic/atonic/drop seizures prominent.
Epilepsy with myoclonic–atonic seizures (EMAtS)	Mandatory myoclonic–atonic seizures, in addition to commonly observed myoclonic, absence, and generalized tonic–clonic seizures, as well as tonic seizures early in the course are exclusionary.	Generalized 2–6 Hz spike- or polyspike-and-wave bursts; background initially normal; GPFA and SSW are exclusionary.	Onset usually occurs at 2–6 years of age (range: 6 months–8 years). Many children remit within a few years and have more favorable cognitive outcomes than those with LGS.	Absence of early tonic seizures and of SSW or GPFA; dominance of myoclonic–atonic seizures; prognosis generally better than LGS.
DEE/EE with spike-and-wave activity during sleep (DEE-SWAS/EE-SWAS)	Presence of mixed focal and generalized seizures. Negative myoclonus and atonic/axial drops may occur, but myoclonic–atonic seizures are not typical.	Marked activation of diffuse spike-and-wave (historical ESES) activity occupying a large proportion of NREM sleep; no mandatory SSW with GPFA.	Childhood-onset; prominent regression of language, cognition, and behavior temporally linked to sleep-activated EEG patterns.	Regression is tightly coupled to near-continuous sleep spike-and-wave activity; tonic seizures and the classic SSW/GPFA triad of LGS are absent.
Dravet syndrome (DS)	Prolonged hemiclonic or generalized tonic–clonic seizures, often febrile, in the first year of life. This is followed by multiple generalized and focal seizure types in later stages, with the absence of myoclonic–atonic seizures.	Generalized and focal spike-wave/poly spike-wave activity; no stereotyped SSW with GPFA.	Onset in infancy (<12 months), frequently with fever sensitivity; developmental slowing emerges over time.	Earlier onset, fever-triggered hemiclonic seizures, and lack of SSW activity or GPFA clearly distinguish DS from LGS.
CLN2 disease (late-infantile neuronal ceroid lipofuscinosis type 2)	Multiple generalized seizure types, often accompanied by an EMAtS-like phenotype, and is characterized by progressive motor and cognitive decline and visual loss.	EEG shows a characteristic low-frequency (1–3 Hz) photoparoxysmal response; not dominated by SSW activity or GPFA.	Onset typically <5 years of age in previously normal or mildly delayed children, accompanied by rapid neurodegeneration.	Progressive ataxia, visual failure, and low-frequency photoparoxysmal responses strongly suggest CLN2 rather than LGS.

Abbreviations: EEG, electroencephalogram; ESES, electrical status epilepticus in sleep; DEE, developmental and epileptic encephalopathy; GPFA, generalized paroxysmal fast activity; LGS, Lennox–Gastaut syndrome; SSW, slow spike–wave; SWAS, spike-and-wave activity during sleep.

**Table 2:** Genes associated with LGS, categorized by function.

Functional Category	Gene	Molecular Mechanism and Phenotypic Description	Relevance to LGS Phenotype	References
Voltage-gated sodium channels	*SCN1A*	Mechanism: LOF (haploinsufficiency) affecting inhibitory interneurons. LGS-related phenotype: Classically causes Dravet syndrome; a subset of patients can show LGS features or progress toward an LGS-spectrum phenotype (tonic/atonic seizures, and SSW/GPFA on EEG).	Demonstrates phenotypic convergence from a distinct genetic epilepsy (Dravet syndrome) toward LGS, supporting the concept of LGS as a shared electroclinical endpoint.	[5,47]
	*SCN2A*	Mechanism: GOF in early-onset epilepsy; loss-of-function is observed more often in later-onset epilepsy/autism. LGS-related phenotype: DEE that may overlap with or evolve into LGS in some individuals; early-onset GOF variants may respond to sodium-channel blockers.	Highlights function-dependent treatment implications and explains why heterogeneous DEEs may fulfill LGS diagnostic criteria over time.	[5,26]
	*SCN8A*	Mechanism: Typically GOF of NaV1.6. LGS-related phenotype: Severe DEE with refractory tonic seizures and generalized epileptiform activity; can meet LGS criteria in some cases.	Provides a mechanistic basis for tonic seizures and generalized EEG patterns characteristic of LGS.	[5,26]
	*SCN10A*	Mechanism: Predicted splice-site variant; role in LGS is still being clarified. LGS-related phenotype: Identified as a (likely) pathogenic LGS-related variant in a cohort of Korean families with LGS/LGS-like epilepsy without structural brain lesions.	Suggests a genetic explanation for non-lesional LGS/LGS-like epilepsy, particularly in population-specific cohorts.	[20]
Voltage-gated potassium channels	*KCNQ2*	Mechanism: LOF (often dominant-negative) of Kv7.2. LGS-related phenotype: Neonatal-onset developmental and epileptic encephalopathy; some cases later display an LGS-spectrum course.	Illustrates a developmental trajectory from neonatal DEE to an LGS-spectrum phenotype.	[26]
	*KCNA2*	Mechanism: LOF or GOF of Kv1.2 depending on the variant. LGS-related phenotype: Epileptic encephalopathy with generalized seizures (often with ataxia); LGS has been reported among the spectrum of presentations.	Supports the clinical heterogeneity of LGS arising from diverse potassium channelopathies.	[26]
	*KCNT1*	Mechanism: GOF of the Na^+^-activated K^+^ channel. LGS-related phenotype: Typically associated with epilepsy of infancy with migrating focal seizures or sleep-related hypermotor epilepsy, but LGS has also been reported; quinidine has been explored for GOF variants.	Emphasizes diagnostic overlap between focal epilepsies and LGS, underscoring the importance of genotype-informed interpretation.	[26,48]
Voltage-gated calcium channels	*CACNA1A*	Mechanism: Variant-dependent; can disrupt CaV2.1 channel function. LGS-related phenotype: Reported in patients with LGS/LGS-like epilepsy (including recessive variants) and included among LGS precision-medicine channelopathies.	Included among channelopathies relevant to precision-medicine approaches in LGS.	[20,26]
	*CACNA2D2*	Mechanism: Biallelic loss-of-function affecting the α2δ-2 calcium-channel auxiliary subunit. LGS-related phenotype: Epileptic encephalopathy with ataxia/cerebellar involvement; reported within the spectrum of disorders that can evolve into LGS.	Indicates that combined epilepsy–ataxia phenotypes may converge toward LGS electroclinical features.	[49]
GABAergic signaling	*GABRA1*	Mechanism: LOF or dominant-negative effects on the GABA-A receptor α1 subunit. LGS-related phenotype: Generalized DEE that can present with LGS seizure types and EEG patterns.	Reinforces impaired inhibitory signaling as a core mechanism underlying LGS network instability.	[26]
	*GABRA2*	Mechanism: Variant-dependent; impaired GABAA receptor α2 function. LGS-related phenotype: Included among GABAergic genes implicated in LGS-spectrum DEE.	Contributes to the GABAergic pathway framework explaining generalized seizure susceptibility in LGS.	[26]
	*GABRB2*	Mechanism: Variant-dependent; GABAA receptor β2 dysfunction. LGS-related phenotype: Included among GABAergic genes implicated in LGS-spectrum DEE.	Further supports inhibitory synapse dysfunction as a shared pathway in LGS-spectrum disorders.	[26]
	*GABRB3*	Mechanism: Loss-of-function/receptor dysfunction. LGS-related phenotype: Recurrent de novo variants reported in epileptic encephalopathies, including LGS; GABRB3 models show LGS-like features.	Experimental models linking receptor dysfunction to LGS-like phenotypes strengthen biological plausibility.	[26,50]
	*SLC6A1*	Mechanism: Loss-of-function of GAT-1 with impaired GABA reuptake (often via trafficking defects). LGS-related phenotype: Can present as DEE/MAE with progression to LGS in some individuals; a functionally characterized LGS-associated missense variant impairs transporter trafficking and function.	Explains clinical progression from MAE/DEE to LGS via impaired inhibitory neurotransmitter homeostasis.	[26,51]
Glutamatergic signaling (NMDA/AMPA)	*GRIN1*	Mechanism: Gain- or loss-of-function NMDA receptor dysfunction. LGS-related phenotype: NMDA receptor DEE with variable seizure types; LGS phenotype reported within the spectrum; selected GOF variants may respond to NMDA antagonists in precision approaches.	Demonstrates how glutamatergic dysfunction contributes to network-level epileptic encephalopathy meeting LGS criteria.	[26]
	*GRIN2A*	Mechanism: Variant-dependent NMDA receptor dysfunction. LGS-related phenotype: Epilepsy-aphasia spectrum and DEE; LGS phenotype reported in some patients.	Highlights overlap between epilepsy–aphasia spectrum and LGS, supporting non–syndrome-specific network vulnerability.	[26]
	*GRIN2B*	Mechanism: Variant-dependent (GOF or LOF). LGS-related phenotype: Severe DEE; West syndrome can evolve to LGS in some cases; precision approaches may consider NMDA modulation depending on functional effect.	Illustrates a developmental continuum from West syndrome to LGS with potential for function-guided therapy.	[26]
	*GRIN2D*	Mechanism: Variant-dependent NMDA receptor dysfunction. LGS-related phenotype: Included among glutamatergic genes implicated in LGS-spectrum DEE.	Supports the role of NMDA-mediated excitatory network dysfunction in LGS.	[26]
	*FRRS1L*	Mechanism: Biallelic LOF affecting AMPA receptor biogenesis. LGS-related phenotype: Epileptic encephalopathy with severe developmental impairment; identified in an LGS/LGS-like cohort (recessive).	Provides evidence that AMPA receptor dysfunction contributes to LGS-like encephalopathy.	[20]
mTOR pathway/mTORopathies	*TSC1*	Mechanism: LOF → constitutive mTOR activation. LGS-related phenotype: Tuberous sclerosis complex (TSC); a common etiology of infantile spasms that may evolve into LGS; mTOR inhibitors can be disease-targeted therapy in TSC-associated epilepsy.	Represents a treatable etiology with well-established progression from infantile spasms to LGS.	[26,52]
	*TSC2*	Mechanism: LOF → constitutive mTOR activation. LGS-related phenotype: As mentioned above; TSC2 variants are frequent in TSC and associated with epilepsy burden, with potential evolution to LGS.	Reinforces mTORopathy as a major pathogenic axis in LGS evolution.	[26,52]
	*DEPDC5*	Mechanism: LOF of the GATOR1 complex → mTOR hyperactivation. LGS-related phenotype: Can cause focal epilepsy and DEE; LGS and infantile epileptic spasms syndrome evolving to LGS have been reported.	Highlights that focal epilepsies may converge into LGS, supporting network-based classification.	[53]
	*MTOR*	Mechanism: Activating (often somatic) variants causing mTORopathies. LGS-related phenotype: Reported in patients with epileptic encephalopathy including LGS; may be associated with cortical malformations and refractory seizures.	Emphasizes the relevance of somatic mosaicism and structural substrates in LGS pathogenesis.	[54]
Presynaptic neurotransmission and vesicle cycling	*STXBP1*	Mechanism: Haploinsufficiency affecting synaptic vesicle exocytosis. LGS-related phenotype: Early infantile epileptic encephalopathy (Ohtahara/West spectrum) with possible evolution to LGS; profound developmental impairment and movement disorders are common.	Represents a canonical developmental trajectory from early DEE to LGS.	[26,48]
	*DNM1*	Mechanism: Dominant-negative effects impairing synaptic vesicle endocytosis. LGS-related phenotype: Severe DEE with infantile spasms that can progress to LGS-spectrum epilepsy.	Supports the concept of synaptic vesicle dysfunction leading to LGS network pathology.	[5,26]
	*SYN1*	Mechanism: Synapsin-I dysfunction affecting neurotransmitter release. LGS-related phenotype: Identified as an epilepsy-related likely pathogenic variant in an LGS/LGS-like cohort.	Provides cohort-based genetic evidence supporting synaptic release defects in LGS.	[20]
	*SYN2*	Mechanism: Synapsin-II dysfunction affecting neurotransmitter release. LGS-related phenotype: Identified in an LGS/LGS-like cohort; contribution may vary by variant.	Highlights genetic heterogeneity underlying LGS-spectrum epilepsy.	[20]
	*DNAJC5*	Mechanism: Presynaptic co-chaperone (CSPα) involved in exocytosis and protein folding. LGS-related phenotype: Identified as an epilepsy-related variant in an LGS/LGS-like cohort.	Suggests impaired presynaptic protein homeostasis as a contributor to LGS.	[20]
Postsynaptic signaling and synaptic scaffolding	*IQSEC2*	Mechanism: LOF in an ARF6 guanine-nucleotide exchange factor regulating excitatory synapses. LGS-related phenotype: X-linked neurodevelopmental disorder with epilepsy; LGS reported, including a multi-generational family with an IQSEC2 variant.	Demonstrates X-linked inheritance patterns in LGS-spectrum disorders.	[20,26,55]
	*SHANK3*	Mechanism: Synaptic scaffolding dysfunction. LGS-related phenotype: Identified as an epilepsy-related variant in an LGS/LGS-like cohort; SHANK3-related disorders can include refractory epilepsy.	Supports postsynaptic structural instability as a substrate for LGS.	[20]
	*MAGI1*	Mechanism: Membrane-associated guanylate kinase scaffolding protein involved in neurite outgrowth. LGS-related phenotype: Candidate neuron-related gene identified in an LGS/LGS-like cohort.	Suggests abnormal synaptic scaffolding contributes to LGS network vulnerability.	[20]
	*NRG2*	Mechanism: Neuregulin signaling affecting neuronal survival and neurite extension. LGS-related phenotype: Candidate neuron-related gene identified in an LGS/LGS-like cohort.	Adds evidence for disrupted neurodevelopmental signaling in LGS.	[20]
	*SSPO*	Mechanism: SCO-spondin; cell adhesion and early neurodevelopment. LGS-related phenotype: Candidate neuron-related gene identified in an LGS/LGS-like cohort (recessive frameshift).	Indicates that early neurodevelopmental adhesion pathways may underlie LGS.	[20]
Chromatin, transcription, and RNA-binding	*CHD2*	Mechanism: Haploinsufficiency of a chromatin remodeler. LGS-related phenotype: One of the more recurrently implicated genes in LGS; often associated with photosensitivity and generalized seizure phenotypes that can meet LGS criteria.	Represents a recurrent genetic contributor linking chromatin remodeling to LGS.	[20,26,56]
	*HDAC4*	Mechanism: Epigenetic dysregulation via altered histone deacetylation. LGS-related phenotype: Reported among genes identified in LGS genetic studies/workshop summaries; associated with neurodevelopmental disorders and epilepsy.	Supports epigenetic dysregulation as a contributing mechanism in LGS.	[5]
	*HNRNPU*	Mechanism: Haploinsufficiency of an RNA-binding protein affecting neuronal gene regulation. LGS-related phenotype: Early-onset epilepsy with developmental impairment; evolution to an LGS-like phenotype has been described.	Highlights RNA-processing defects in LGS-spectrum epilepsy.	[57]
	*MEF2C*	Mechanism: Haploinsufficiency (often via 5q14.3 disruption) affecting neuronal transcriptional programs. LGS-related phenotype: LGS reported in a case with chromothripsis involving 5q14.3; MEF2C region disruption is a known cause of neurodevelopmental epilepsy.	Demonstrates transcriptional dysregulation as a substrate for LGS.	[58]
Kinase and intracellular signaling	*CDKL5*	Mechanism: Loss of kinase activity. LGS-related phenotype: CDKL5 deficiency disorder with early-onset, drug-resistant seizures; LGS phenotype can develop over time.	Supports kinase-related DEE as an evolving LGS phenotype.	[5,26,48,59]
	*ALG13*	Mechanism: X-linked DEE; variant-dependent mechanisms affecting glycosylation/translation-associated pathways (mechanism still under investigation). LGS-related phenotype: Reported in genetic studies of LGS and in DEE evolving to LGS-spectrum epilepsy.	Highlights metabolic–signaling cross-talk in LGS development.	[5,60]
	*PPP3CA*	Mechanism: De novo variants disrupt calcineurin signaling. LGS-related phenotype: Severe neurodevelopmental disorder with refractory seizures; LGS phenotype is among reported electroclinical diagnoses.	Indicates calcium-dependent intracellular signaling in LGS pathophysiology.	[61]
Mitochondrial/metabolic	*SLC25A39*	Mechanism: Mitochondrial carrier; potential role in heme/iron metabolism (under investigation). LGS-related phenotype: Candidate de novo variant identified in an LGS/LGS-like cohort; currently limited evidence and may act as a risk/modifier gene.	Suggests mitochondrial vulnerability as a modifier in LGS.	[20]
	*TBC1D8*	Mechanism: Vesicle-mediated transport (TBC1 domain family). LGS-related phenotype: Candidate de novo variant identified in an LGS/LGS-like cohort; evidence remains limited.	Supports polygenic or modifier contributions to LGS.	[20]
	*ADSL*	Mechanism: Adenylosuccinate lyase deficiency affecting purine metabolism. LGS-related phenotype: Identified among pathogenic/likely pathogenic variants in children with refractory epilepsy, including cases evolving from West syndrome to LGS.	Illustrates metabolic epilepsies progressing toward LGS.	[48]
Somatic mosaicism/glycosylation	*SLC35A2*	Mechanism: Somatic (brain) variants affecting UDP-galactose transport and N-glycosylation; associated with MOGHE. LGS-related phenotype: Case series show frequent LGS and/or evolution from infantile spasms to LGS in patients with brain somatic SLC35A2 variants.	Strongly links somatic mosaicism to LGS evolution.	[62]
Neurodegeneration/NBIA and related etiologies	*WDR45*	Mechanism: LOF causing beta-propeller protein-associated neurodegeneration. LGS-related phenotype: Seizures with EEG patterns consistent with LGS have been reported in WDR45-related disease.	Demonstrates that neurodegenerative disorders may manifest LGS electroclinical features.	[5,63]
Other established genetic etiologies	*FOXG1*	Mechanism: Neurodevelopmental transcription factor dysfunction. LGS-related phenotype: A documented case report describes LGS in FOXG1-related disorder; FOXG1 variants can cause severe DEE with infantile spasms evolving to LGS.	Illustrates rare but definitive genetic causes of LGS.	[60,64]
	*WWOX*	Mechanism: Biallelic loss-of-function causing WWOX-related DEE. LGS-related phenotype: Reported across WWOX encephalopathy cases, including infantile spasms with later evolution to LGS-spectrum epilepsy.	Reinforces infantile spasms as a key precursor to LGS.	[65,66]

Abbreviations: DEE, developmental and epileptic encephalopathy; SSW, slow spike-and-wave; GPFA, generalized paroxysmal fast activity; GOF, gain-of-function; LOF, loss-of-function; MOGHE, mild malformation of cortical development with oligodendroglial hyperplasia in epilepsy; LGS, Lennox–Gastaut Syndrome; mTOR, mechanistic target of rapamycin; EEG, electroencephalogram; NMDA, N-methyl-D-aspartate; AMPA, α-amino-3-hydroxy-5-methyl-4-isoxazolepropionic acid.

## Data Availability

No new data were created or analyzed in this study. Data sharing is not applicable to this article.

## Abbreviations

AMPA	α-Amino-3-hydroxy-5-methyl-4-isoxazolepropionic acid
ASM	Anti-seizure medication
CB1	Cannabinoid receptor type 1
CB2	Cannabinoid receptor type 2
cKD	Classical ketogenic diet
DBS	Deep brain stimulation
DEE	Developmental and epileptic encephalopathy
DS	Dravet syndrome
EEG	Electroencephalogram
EIEE	Early infantile epileptic encephalopathy
EMAtS	Epilepsy with myoclonic–atonic seizures
ESES	Electrical status epilepticus in sleep
fMRI	Functional magnetic resonance imaging
GABA	γ-Aminobutyric acid
GPFA	Generalized paroxysmal fast activity
ILAE	International League Against Epilepsy
IESS	Infantile epileptic spasms syndrome
LGS	Lennox–Gastaut syndrome
MAD	Modified Atkins diet
mTOR	Mechanistic target of rapamycin
NMDA	N-Methyl-D-aspartate
RNS	Responsive neurostimulation

## References

[1] Cerulli Irelli E., Petrungaro A., Pastorino G.M.G., Mazzeo A., Morano A., Casciato S., Salati E., Operto F.F., Giallonardo A.T., Di Gennaro G. (2024). Long-term outcomes and adaptive behavior in adult patients with Lennox-Gastaut syndrome. Epilepsia Open.

[2] Gelisse P., Crespel A., Genton P., Dravet C. (2025). History of Lennox-Gastaut Syndrome: An electro-clinical voyage in search of an epileptic syndrome. Seizure.

[3] Sullivan J., Benitez A., Roth J., Andrews J.S., Shah D., Butcher E., Jones A., Cross J.H. (2024). A systematic literature review on the global epidemiology of Dravet syndrome and Lennox-Gastaut syndrome: Prevalence, incidence, diagnosis, and mortality. Epilepsia.

[4] Guerrini R., Conti V., Mantegazza M., Balestrini S., Galanopoulou A.S., Benfenati F. (2023). Developmental and epileptic encephalopathies: From genetic heterogeneity to phenotypic continuum. Physiol. Rev..

[5] Riva A., D’Onofrio G., Amadori E., Arzimanoglou A., Auvin S., Bagnasco I., Barabino P., Biagioli V., Brambilla I., Cangemi G. (2025). Lennox-Gastaut syndrome unveiled: Advancing diagnosis, therapies, and advocacy-insights from the Genoa International Workshop. Epilepsia.

[6] Beniczky S., Trinka E., Wirrell E., Abdulla F., Al Baradie R., Alonso Vanegas M., Auvin S., Singh M.B., Blumenfeld H., Bogacz Fressola A. (2025). Updated classification of epileptic seizures: Position paper of the International League Against Epilepsy. Epilepsia.

[7] Specchio N., Wirrell E.C., Scheffer I.E., Nabbout R., Riney K., Samia P., Guerreiro M., Gwer S., Zuberi S.M., Wilmshurst J.M. (2022). International League Against Epilepsy classification and definition of epilepsy syndromes with onset in childhood: Position paper by the ILAE Task Force on Nosology and Definitions. Epilepsia.

[8] Auvin S., Arzimanoglou A., Falip M., Striano P., Cross J.H. (2025). Refining management strategies for Lennox-Gastaut syndrome: Updated algorithms and practical approaches. Epilepsia Open.

[9] Amrutkar C.V., Lui F. (2025). Lennox-Gastaut Syndrome. StatPearls.

[10] Strzelczyk A., Zuberi S.M., Striano P., Rosenow F., Schubert-Bast S. (2023). The burden of illness in Lennox-Gastaut syndrome: A systematic literature review. Orphanet J. Rare Dis..

[11] Nelson J.A., Knupp K.G. (2023). Lennox-Gastaut Syndrome: Current Treatments, Novel Therapeutics, and Future Directions. Neurotherapeutics.

[12] Devinsky O., Patel A.D., Cross J.H., Villanueva V., Wirrell E.C., Privitera M., Greenwood S.M., Roberts C., Checketts D., VanLandingham K.E. (2018). Effect of Cannabidiol on Drop Seizures in the Lennox-Gastaut Syndrome. N. Engl. J. Med..

[13] Cho S., Makhalova J., Medina Villalon S., Villeneuve N., Trebuchon A., Krouma M., Scavarda D., Lepine A., Milh M., Carron R. (2025). Stereoelectroencephalographic exploration and surgical outcome in Lennox-Gastaut syndrome. Epilepsia.

[14] Nizami F.M., Trivedi S., Kalita J. (2024). A systematic review of electroencephalographic findings in Lennox-Gastaut syndrome. Epilepsy Res..

[15] Na J.H., Lee Y.M. (2024). Therapeutic outcome of patients with Lennox-Gastaut syndrome with mitochondrial respiratory chain complex I deficiency. Front. Neurol..

[16] Berg A.T., Gaebler-Spira D., Wilkening G., Zelko F., Knupp K., Dixon-Salazar T., Villas N., Meskis M.A., Harwell V., Thompson T. (2020). Nonseizure consequences of Dravet syndrome, KCNQ2-DEE, KCNB1-DEE, Lennox-Gastaut syndrome, ESES: A functional framework. Epilepsy Behav..

[17] Asadi-Pooya A.A., Bazrafshan M., Farazdaghi M. (2021). Long-term medical and social outcomes of patients with Lennox-Gastaut syndrome. Epilepsy Res..

[18] Specchio N., Curatolo P. (2021). Developmental and epileptic encephalopathies: What we do and do not know. Brain.

[19] Zhou Z., Jiao X., Gong P., Niu Y., Xu Z., Zhang G., Zhang Y., Qin J., Yang Z. (2024). Clinical features and underlying etiology of children with Lennox-Gastaut syndrome. J. Neurol..

[20] Yang J.O., Choi M.H., Yoon J.Y., Lee J.J., Nam S.O., Jun S.Y., Kwon H.H., Yun S., Jeon S.J., Byeon I. (2021). Characteristics of Genetic Variations Associated With Lennox-Gastaut Syndrome in Korean Families. Front. Genet..

[21] Qaiser F., Sadoway T., Yin Y., Zulfiqar Ali Q., Nguyen C.M., Shum N., Backstrom I., Marques P.T., Tabarestani S., Munhoz R.P. (2021). Genome sequencing identifies rare tandem repeat expansions and copy number variants in Lennox-Gastaut syndrome. Brain Commun..

[22] Warren A.E.L., Patel A.D., Helen Cross J., Clarke D.F., Dalic L.J., Grinspan Z.M., Conecker G., Knowles J.K. (2025). Mobilizing a New Era in Lennox-Gastaut Syndrome Treatment and Prevention. Epilepsy Curr..

[23] Montebello G., Di Giovanni G. (2025). Dysregulation of the Cannabinoid System in Childhood Epilepsy: From Mechanisms to Therapy. Int. J. Mol. Sci..

[24] Moon J.U., Cho K.O. (2021). Current Pharmacologic Strategies for Treatment of Intractable Epilepsy in Children. Int. Neurourol. J..

[25] Ng A.C., Chahine M., Scantlebury M.H., Appendino J.P. (2024). Channelopathies in epilepsy: An overview of clinical presentations, pathogenic mechanisms, and therapeutic insights. J. Neurol..

[26] Samanta D. (2025). Precision Therapeutics in Lennox-Gastaut Syndrome: Targeting Molecular Pathophysiology in a Developmental and Epileptic Encephalopathy. Children.

[27] Epi K.C., Epilepsy Phenome/Genome P., Allen A.S., Berkovic S.F., Cossette P., Delanty N., Dlugos D., Eichler E.E., Epstein M.P., Glauser T. (2013). De novo mutations in epileptic encephalopathies. Nature.

[28] Abramov D., Guiberson N.G.L., Burre J. (2021). STXBP1 encephalopathies: Clinical spectrum, disease mechanisms, and therapeutic strategies. J. Neurochem..

[29] Dalic L.J., Warren A.E.L., Spiegel C., Thevathasan W., Roten A., Bulluss K.J., Archer J.S. (2022). Paroxysmal fast activity is a biomarker of treatment response in deep brain stimulation for Lennox-Gastaut syndrome. Epilepsia.

[30] Siniatchkin M., Coropceanu D., Moeller F., Boor R., Stephani U. (2011). EEG-fMRI reveals activation of brainstem and thalamus in patients with Lennox-Gastaut syndrome. Epilepsia.

[31] Warren A.E.L., Butson C.R., Hook M.P., Dalic L.J., Archer J.S., Macdonald-Laurs E., Schaper F., Hart L.A., Singh H., Johnson L. (2024). Targeting thalamocortical circuits for closed-loop stimulation in Lennox-Gastaut syndrome. Brain Commun..

[32] Lindquist B.E., Timbie C., Voskobiynyk Y., Paz J.T. (2023). Thalamocortical circuits in generalized epilepsy: Pathophysiologic mechanisms and therapeutic targets. Neurobiol. Dis..

[33] Johnson G.W., Doss D.J., Englot D.J. (2022). Network dysfunction in pre and postsurgical epilepsy: Connectomics as a tool and not a destination. Curr. Opin. Neurol..

[34] Viswanathan S., Oliver K.L., Regan B.M., Schneider A.L., Myers C.T., Mehaffey M.G., LaCroix A.J., Antony J., Webster R., Cardamone M. (2024). Solving the Etiology of Developmental and Epileptic Encephalopathy with Spike-Wave Activation in Sleep (D/EE-SWAS). Ann. Neurol..

[35] Medyanik A.D., Anisimova P.E., Kustova A.O., Tarabykin V.S., Kondakova E.V. (2025). Developmental and Epileptic Encephalopathy: Pathogenesis of Intellectual Disability Beyond Channelopathies. Biomolecules.

[36] Scott R.C., Hsieh J., McTague A., Mahoney J.M., Christian-Hinman C.A. (2025). Merritt-Putnam Symposium | Developmental and Epileptic Encephalopathies-Current Concepts and Novel Approaches. Epilepsy Curr..

[37] He Q., Zhuang J., Wen Q., Li Z., Wang D., Sun X., Xie Y. (2022). Epilepsy Combined With Multiple Gene Heterozygous Mutation. Front. Pediatr..

[38] Strzelczyk A., Schubert-Bast S. (2021). Expanding the Treatment Landscape for Lennox-Gastaut Syndrome: Current and Future Strategies. CNS Drugs.

[39] Oliver K.L., Scheffer I.E., Bennett M.F., Grinton B.E., Bahlo M., Berkovic S.F. (2023). Genes4Epilepsy: An epilepsy gene resource. Epilepsia.

[40] Romero Mila B., Liu V.B., Smith R.J., Hu D.K., Benneian N.A., Hussain S.A., Steenari M., Phillips D., Adams D., Skora C. (2025). EEG functional connectivity as a marker of evolution from infantile epileptic spasms syndrome to Lennox-Gastaut syndrome. Epilepsia.

[41] Montouris G., Aboumatar S., Burdette D., Kothare S., Kuzniecky R., Rosenfeld W., Chung S. (2020). Expert opinion: Proposed diagnostic and treatment algorithms for Lennox-Gastaut syndrome in adult patients. Epilepsy Behav..

[42] Freibauer A.E., RamachandranNair R., Jain P., Jones K.C., Whitney R. (2023). The genetic landscape of developmental and epileptic encephalopathy with spike-and-wave activation in sleep. Seizure.

[43] Borowicz-Reutt K., Czernia J., Krawczyk M. (2023). Genetic Background of Epilepsy and Antiepileptic Treatments. Int. J. Mol. Sci..

[44] Bernasconi A., Cendes F., Theodore W.H., Gill R.S., Koepp M.J., Hogan R.E., Jackson G.D., Federico P., Labate A., Vaudano A.E. (2019). Recommendations for the use of structural magnetic resonance imaging in the care of patients with epilepsy: A consensus report from the International League Against Epilepsy Neuroimaging Task Force. Epilepsia.

[45] Sheidley B.R., Malinowski J., Bergner A.L., Bier L., Gloss D.S., Mu W., Mulhern M.M., Partack E.J., Poduri A. (2022). Genetic testing for the epilepsies: A systematic review. Epilepsia.

[46] Na J.H., Shin S., Yang D., Kim B., Kim H.D., Kim S., Lee J.S., Choi J.R., Lee S.T., Kang H.C. (2020). Targeted gene panel sequencing in early infantile onset developmental and epileptic encephalopathy. Brain Dev..

[47] He N., Lin Z.J., Wang J., Wei F., Meng H., Liu X.R., Chen Q., Su T., Shi Y.W., Yi Y.H. (2019). Evaluating the pathogenic potential of genes with de novo variants in epileptic encephalopathies. Genet. Med..

[48] Liu J., Tong L., Song S., Niu Y., Li J., Wu X., Zhang J., Zai C.C., Luo F., Wu J. (2018). Novel and de novo mutations in pediatric refractory epilepsy. Mol. Brain.

[49] Pippucci T., Parmeggiani A., Palombo F., Maresca A., Angius A., Crisponi L., Cucca F., Liguori R., Valentino M.L., Seri M. (2013). A novel null homozygous mutation confirms CACNA2D2 as a gene mutated in epileptic encephalopathy. PLoS ONE.

[50] Papandreou A., McTague A., Trump N., Ambegaonkar G., Ngoh A., Meyer E., Scott R.H., Kurian M.A. (2016). GABRB3 mutations: A new and emerging cause of early infantile epileptic encephalopathy. Dev. Med. Child. Neurol..

[51] Cai K., Wang J., Eissman J., Wang J., Nwosu G., Shen W., Liang H.C., Li X.J., Zhu H.X., Yi Y.H. (2019). A missense mutation in SLC6A1 associated with Lennox-Gastaut syndrome impairs GABA transporter 1 protein trafficking and function. Exp. Neurol..

[52] Jeong A., Wong M. (2018). Targeting the Mammalian Target of Rapamycin for Epileptic Encephalopathies and Malformations of Cortical Development. J. Child. Neurol..

[53] Zhao T., Chen F., Cao B., Yang L., Yin J., Li Y., Feng F. (2025). Case Report: Unraveling clinical heterogeneity in DEPDC5-related epilepsy: A genotype-phenotype correlation study in eight pediatric cases. Front. Neurosci..

[54] Park S.M., Lim J.S., Ramakrishina S., Kim S.H., Kim W.K., Lee J., Kang H.C., Reiter J.F., Kim D.S., Kim H.H. (2018). Brain Somatic Mutations in MTOR Disrupt Neuronal Ciliogenesis, Leading to Focal Cortical Dyslamination. Neuron.

[55] Choi M.H., Yang J.O., Min J.S., Lee J.J., Jun S.Y., Lee Y.J., Yoon J.Y., Jeon S.J., Byeon I., Kang J.W. (2020). A Novel X-Linked Variant of IQSEC2 is Associated with Lennox-Gastaut Syndrome and Mild Intellectual Disability in Three Generations of a Korean Family. Genet. Test. Mol. Biomark..

[56] Asadi-Pooya A.A. (2018). Lennox-Gastaut syndrome: A comprehensive review. Neurol. Sci..

[57] Bramswig N.C., Lüdecke H.J., Hamdan F.F., Altmüller J., Beleggia F., Elcioglu N.H., Freyer C., Gerkes E.H., Demirkol Y.K., Knupp K.G. (2017). Heterozygous HNRNPU variants cause early onset epilepsy and severe intellectual disability. Hum. Genet..

[58] Corriveau M.L., Korb J.C., Michener S.L., Owen N.M., Wilson E.L., Kubala J., Turner A., Takacs D.S., Potocki L., Swann J.W. (2025). De Novo Chromosomes 3q and 5q Chromothripsis Leads to a 5q14.3 Microdeletion Syndrome Presentation: Case Report and Review of the Literature. Am. J. Med. Genet. A.

[59] Kalinowska-Doman A., Strzelczyk A., Paprocka J. (2025). Antiseizure medications in CDKL5 encephalopathy-systematic review. Seizure.

[60] Mastrangelo M. (2017). Lennox-Gastaut Syndrome: A State of the Art Review. Neuropediatrics.

[61] Myers C.T., Stong N., Mountier E.I., Helbig K.L., Freytag S., Sullivan J.E., Ben Zeev B., Nissenkorn A., Tzadok M., Heimer G. (2017). De Novo Mutations in PPP3CA Cause Severe Neurodevelopmental Disease with Seizures. Am. J. Hum. Genet..

[62] Bonduelle T., Hartlieb T., Baldassari S., Sim N.S., Kim S.H., Kang H.C., Kobow K., Coras R., Chipaux M., Dorfmüller G. (2021). Frequent SLC35A2 brain mosaicism in mild malformation of cortical development with oligodendroglial hyperplasia in epilepsy (MOGHE). Acta Neuropathol. Commun..

[63] Gregory A., Kurian M.A., Haack T., Hayflick S.J., Hogarth P., Adam M.P., Bick S., Mirzaa G.M., Pagon R.A., Wallace S.E., Amemiya A. (1993). Beta-Propeller Protein-Associated Neurodegeneration. GeneReviews^®^.

[64] Terrone G., Bienvenu T., Germanaud D., Barthez-Carpentier M.A., Diebold B., Delanoe C., Passemard S., Auvin S. (2014). A case of Lennox-Gastaut syndrome in a patient with FOXG1-related disorder. Epilepsia.

[65] Ehaideb S.N., Al-Bu Ali M.J., Al-Obaid J.J., Aljassim K.M., Alfadhel M. (2018). Novel Homozygous Mutation in the WWOX Gene Causes Seizures and Global Developmental Delay: Report and Review. Transl. Neurosci..

[66] Burgess R., Wang S., McTague A., Boysen K.E., Yang X., Zeng Q., Myers K.A., Rochtus A., Trivisano M., Gill D. (2019). The Genetic Landscape of Epilepsy of Infancy with Migrating Focal Seizures. Ann. Neurol..

[67] Samanta D., Nath M. (2025). Current and emerging pharmacotherapies in Lennox-Gastaut syndrome. Expert. Opin. Pharmacother..

[68] Samanta D., Bhalla S., Bhatia S., Fine A.L., Haridas B., Karakas C., Keator C.G., Koh H.Y., Perry M.S., Stafstrom C.E. (2025). Antiseizure medications for Lennox-Gastaut Syndrome: Comprehensive review and proposed consensus treatment algorithm. Epilepsy Behav..

[69] Roberti R., Riva A., Striano P., Russo E. (2025). Drug-drug interaction between anti-seizure medications in Dravet syndrome and Lennox-Gastaut syndrome. Expert. Opin. Drug Metab. Toxicol..

[70] Samanta D. (2025). Perampanel, Brivaracetam, Cenobamate, Stiripentol, and Ganaxolone in Lennox-Gastaut Syndrome: A Comprehensive Narrative Review. J. Clin. Med..

[71] Pong A.W. (2025). Expanding the toolkit: An update on the evolution of new therapies for Lennox-Gastaut Syndrome. Semin. Pediatr. Neurol..

[72] Wechsler R.T., Burdette D.E., Gidal B.E., Hyslop A., McGoldrick P.E., Thiele E.A., Valeriano J. (2024). Consensus panel recommendations for the optimization of EPIDIOLEX(R) treatment for seizures associated with Lennox-Gastaut syndrome, Dravet syndrome, and tuberous sclerosis complex. Epilepsia Open.

[73] Na J.-H., Lee Y.-M. (2025). Pharmacological and Pharmacokinetic Profile of Cannabidiol in Human Epilepsy: A Review of Metabolism, Therapeutic Drug Monitoring, and Interactions with Antiseizure Medications. Biomolecules.

[74] Abbotts K.S.S., Ewell T.R., Butterklee H.M., Bomar M.C., Akagi N., Dooley G.P., Bell C. (2022). Cannabidiol and Cannabidiol Metabolites: Pharmacokinetics, Interaction with Food, and Influence on Liver Function. Nutrients.

[75] Samanta D., Naik S. (2025). Efficacy and safety of cenobamate in developmental and epileptic encephalopathies: A systematic review and meta-analysis. Seizure.

[76] Pong A.W., Wehland M., Klein P. (2025). Efficacy, safety and tolerability of adjunctive cenobamate in pediatric and adult patients with Lennox Gastaut Syndrome. Epilepsy Behav..

[77] Skrobas U., Duda P., Brylinski L., Drozak P., Pelczar M., Rejdak K. (2022). Ketogenic Diets in the Management of Lennox-Gastaut Syndrome-Review of Literature. Nutrients.

[78] Martin-McGill K.J., Bresnahan R., Levy R.G., Cooper P.N. (2020). Ketogenic diets for drug-resistant epilepsy. Cochrane Database Syst. Rev..

[79] Duda P., Granat M., Czuczwar S.J., Miziak B. (2025). Non-Pharmacological Treatment Methods of Lennox-Gastaut Syndrome-Review of the Literature. Biomedicines.

[80] Na J.H., Jung D.E., Kang H.J., Kang H.C., Kim H.D. (2022). Treatment strategies for Lennox-Gastaut syndrome: Outcomes of multimodal treatment approaches. Ther. Adv. Neurol. Disord..

[81] Na J.H., Lee H., Lee Y.M. (2025). Clinical Efficacy and Safety of the Ketogenic Diet in Patients with Genetic Confirmation of Drug-Resistant Epilepsy. Nutrients.

[82] Samanta D. (2025). Cognitive and behavioral impact of antiseizure medications, neuromodulation, ketogenic diet, and surgery in Lennox-Gastaut syndrome: A comprehensive review. Epilepsy Behav..

[83] Samanta D., Aungaroon G., Fine A.L., Karakas C., Chiu M.Y., Jain P., Seinfeld S., Knowles J.K., Mohamed I.S., Stafstrom C.E. (2025). Neuromodulation Strategies in Lennox-Gastaut Syndrome: Practical Clinical Guidance from the Pediatric Epilepsy Research Consortium. Epilepsy Res..

[84] Ferreira Soares D., Pires de Aguiar P.H. (2023). Callosotomy vs Vagus Nerve Stimulation in the Treatment of Lennox-Gastaut Syndrome: A Systematic Review With Meta-Analysis. Neuromodulation.

[85] Lyons P., Wheless J., Verner R., Ferreira J., Liow K., Valeriano J., Motamedi G., Giannicola G., Nichol K. (2025). Vagus nerve stimulation in Lennox-Gastaut syndrome: Twenty-four-month data and experience from the CORE-VNS study. Epilepsia.

[86] Gouveia F.V., Warsi N.M., Suresh H., Matin R., Ibrahim G.M. (2024). Neurostimulation treatments for epilepsy: Deep brain stimulation, responsive neurostimulation and vagus nerve stimulation. Neurotherapeutics.

[87] Al-Ramadhani R., Hect J.L., Abel T.J. (2024). The changing landscape of palliative epilepsy surgery for Lennox Gastaut Syndrome. Front. Neurol..

[88] Warren A.E.L., Xu A., Barros Guinle M.I., Johnstone T., Teeyagura P., Solidum R., Hyslop A., Kim H., Grant G., Parker J.J. (2025). Optimal Stimulation of the Thalamic Centromedian Nucleus in Children with Lennox–Gastaut Syndrome: Patient Series. J. Neurosurg. Case Lessons.

[89] Hagen C.W., Keator C.G. (2025). Surgical management of Lennox-Gastaut syndrome: A focused update on resective surgery and corpus callosotomy. Semin. Pediatr. Neurol..

[90] Kang J.W., Eom S., Hong W., Kwon H.E., Park S., Ko A., Kang H.C., Lee J.S., Lee Y.M., Kim D.S. (2018). Long-term Outcome of Resective Epilepsy Surgery in Patients With Lennox-Gastaut Syndrome. Pediatrics.

[91] Roth J., Bergman L., Weil A.G., Brunette-Clement T., Weiner H.L., Treiber J.M., Shofty B., Cukiert A., Cukiert C.M., Tripathi M. (2023). Added value of corpus callosotomy following vagus nerve stimulation in children with Lennox-Gastaut syndrome: A multicenter, multinational study. Epilepsia.

[92] Lam S., Rosenman M., Dixon-Salazar T., Knupp K.G., Thio L.L., Abel T.J., Welch W.P., Reed L., Randle S.C., Garcia-Sosa R. (2025). Comparative effectiveness of epilepsy surgery versus additional anti-seizure medications for Lennox-Gastaut syndrome: Study protocol for a multicenter, mixed-methods study. Front. Neurol..

[93] Samanta D., Bhatia S., Hunter S.E., Rao C.K., Xiong K., Karakas C., Reeders P.C., Erdemir G., Sattar S., Axeen E. (2025). Current and Emerging Precision Therapies for Developmental and Epileptic Encephalopathies. Pediatr. Neurol..

[94] Samanta D., Naik S. (2025). Seizure-type-specific treatment responses in Lennox-Gastaut Syndrome: A comprehensive review of pharmacological, neuromodulatory, dietary, and surgical therapies. Epilepsy Behav..

[95] Samanta D., Jain P., Cunningham J., Arya R. (2025). Comparative efficacy of neuromodulation therapies in Lennox-Gastaut syndrome: A systematic review and meta-analysis of vagus nerve stimulation, deep brain stimulation, and responsive neurostimulation. Epilepsia.

[96] Chiu M.Y., Keator C.G., Warren A.E.L., Knowles J.K., Samanta D., Dixon-Salazar T., Koh H.Y., Seinfeld S.A., Paolicchi J., Vidaurre J. (2025). Current practices and trends in surgical decision-making for children with Lennox-Gastaut syndrome: A cross-sectional survey by the Pediatric Epilepsy Research Consortium. Epilepsia Open.

[97] Auvin S., Nortvedt C., Fuller D.S., Sahebkar F. (2023). Seizure-free days as a novel outcome in patients with Lennox-Gastaut syndrome: Post hoc analysis of patients receiving cannabidiol in two randomized controlled trials. Epilepsia.

[98] Gelisse P., Crespel A., Genton P., Dravet C. (2025). History of lennox-gastaut syndrome: Sixty years of advancements in therapeutic practices. Seizure.

[99] Zhu Z., Zhang Z., Xiao W., Wang C., Liang R. (2025). Efficacy and safety of pharmacological and non-pharmacological therapies in Lennox-Gastaut syndrome: A systematic review and network meta-analysis. Front. Pharmacol..

[100] Zhang L., Jiang N., Wang C. (2025). Efficacy, safety, and tolerability of soticlestat (TAK-935) as adjunctive therapy in pediatric patients with dravet syndrome and Lennox-Gastaut syndrome: A meta-analysis of 3 randomized controlled trials. Front. Pharmacol..

